# The Cancer Cell Dissemination Machinery as an Immunosuppressive Niche: A New Obstacle Towards the Era of Cancer Immunotherapy

**DOI:** 10.3389/fimmu.2021.654877

**Published:** 2021-04-13

**Authors:** Saeed Asiry, Gina Kim, Panagiota S. Filippou, Luis Rivera Sanchez, David Entenberg, Douglas K. Marks, Maja H. Oktay, George S. Karagiannis

**Affiliations:** ^1^Department of Pathology, Montefiore Medical Center, Albert Einstein College of Medicine, New York City, NY, United States; ^2^Department of Surgery, Montefiore Medical Center, Albert Einstein College of Medicine, New York City, NY, United States; ^3^School of Health and Life Sciences, Teesside University, Middlesbrough, United Kingdom; ^4^National Horizons Centre, Teesside University, Darlington, United Kingdom; ^5^Department of Anatomy and Structural Biology, Albert Einstein College of Medicine, New York City, NY, United States; ^6^Gruss-Lipper Biophotonics Center, Albert Einstein College of Medicine, New York City, NY, United States; ^7^Integrated Imaging Program, Albert Einstein College of Medicine, New York City, NY, United States; ^8^Department of Medicine, NYU Long Island School of Medicine, Mineola, NY, United States

**Keywords:** cancer immunotherapy, tumor microenvironment, endothelial anergy, lymphocyte exclusion, lymphocyte exhaustion, metastasis, macrophages, T cells

## Abstract

Although cancer immunotherapy has resulted in unpreceded survival benefits to subsets of oncology patients, accumulating evidence from preclinical animal models suggests that the immunosuppressive tumor microenvironment remains a detrimental factor limiting benefit for many patient subgroups. Recent efforts on lymphocyte-mediated immunotherapies are primarily focused on eliminating cancer foci at primary and metastatic sites, but few studies have investigated the impact of these therapies on the highly complex process of cancer cell dissemination. The metastatic cascade involves the directional streaming of invasive/migratory tumor cells toward specialized blood vessel intravasation gateways, called TMEM doorways, to the peripheral circulation. Importantly, this process occurs under the auspices of a specialized tumor microenvironment, herewith referred to as “Dissemination Trajectory”, which is supported by an ample array of tumor-associated macrophages (TAMs), skewed towards an M2-like polarization spectrum, and which is also vital for providing microenvironmental cues for cancer cell invasion, migration and stemness. Based on pre-existing evidence from preclinical animal models, this article outlines the hypothesis that dissemination trajectories do not only support the metastatic cascade, but also embody immunosuppressive niches, capable of providing transient and localized immunosubversion cues to the migratory/invasive cancer cell subpopulation while in the act of departing from a primary tumor. So long as these dissemination trajectories function as “immune deserts”, the migratory tumor cell subpopulation remains efficient in evading immunological destruction and seeding metastatic sites, despite administration of cancer immunotherapy and/or other cytotoxic treatments. A deeper understanding of the molecular and cellular composition, as well as the signaling circuitries governing the function of these dissemination trajectories will further our overall understanding on TAM-mediated immunosuppression and will be paramount for the development of new therapeutic strategies for the advancement of optimal cancer chemotherapies, immunotherapies, and targeted therapies.

## Introduction

Molecular investigations of the intricate and reciprocal interactions between tumor and immune cells have been at the frontier of cancer research in the past decade, a trend that will likely continue given the recent development of highly effective cancer immunotherapies (1–7). In general, antitumor immunity is strongly reliant on the trafficking of CD8^+^ T cells in both primary and metastatic tumor microenvironments (TMEs) and can be characterized as a highly dynamic and tightly regulated process (8–10). There is abundant preclinical and clinical evidence that the presence of tumor-infiltrating lymphocytes (TILs) correlates with favorable clinical outcomes (11–17), but contradictory results have also been reported (18, 19). Moreover, recent studies have demonstrated that the spatial distribution patterns of TILs within the tumor microenvironment may play an even more drastic role in determining the prognostic outcome, than the density of TILs alone (20–22). In yet other studies, the co-assessment of immune cell signatures related to specific functional status (or subtypes) of TILs may be critical for a more accurate assessment of prognostic outcomes (23–30). These observations collectively suggest that T cell trafficking into the TME is one of the critical aspects of antitumor immunity. The overall immune landscape in the TME is therefore a key determinant for the efficiency of CD8^+^ T cell-mediated antitumor immunity in either natural, induced or engineered immune responses.

The intricate relationship between immune and cancer cells in the context of tumor development and progression has long been recognized (31). Since the initial proposal of the cancer immunosurveillance theory (32, 33), numerous immunotherapies have been developed including monoclonal antibodies (34, 35), chimeric antigen receptor (CAR) T cells (36–38), and tumor vaccines (39, 40). Despite the success, which is primarily seen in hematological malignancies, such as in leukemia and lymphoma (41, 42), the efficacy of these treatment modalities has been less dramatic in solid tumors, such as in breast, colorectal, and prostate cancers (31, 43). The lack of promising outcomes in these solid tumor types is likely a multifactorial and cumulative result arising not only from intrinsic defects of antitumor immunity, but also from the intricate relationships among tumor cells, immune cells, and their surrounding microenvironment, which can obfuscate these antitumoral immune responses (44–46). Although the majority of these mechanisms will not be detailed as they are beyond the scope of the current perspective, here we focus on the emerging roles of the tumor-infiltrating myeloid cell population in limiting antitumor CD8^+^ T cell responses.

A plethora of terminally differentiated myeloid cells and/or their immature counterparts, including monocytes, macrophages, neutrophils, and myeloid derived suppressor cells (MDSCs) among others, have been identified in the tumor stroma, whereby they conspire with tumor cells to promote the acquisition of metastatic hallmarks (47–52). In this heterogeneous landscape, a flurry of proangiogenic and proinflammatory cytokines (VEGF, IL6, etc.) rising from hypoxic and acidic microenvironments instigate myeloid cell infiltration and activation (53–60). There is now ample evidence that this myeloid cell-dominated milieu constitutes a rather inhospitable and antagonistic microenvironment for T cell trafficking and further promotes T cell exhaustion and deactivation (61–68). As such, the latest advances in immunotherapy have been directed at overcoming the immunosuppressive mechanisms within the tumor microenvironment, with a special focus on counteracting the function of protumoral myeloid cell populations (31).

It is undeniable that modern immunotherapies, including immune checkpoint blockade (anti-PDL1, anti-CTLA4, etc.) and adoptive transfer of genetically engineered T cells to express a receptor that is specific for a tumor antigen have revolutionized cancer treatment (41, 69–72). However, most such studies have primarily evaluated cancer cell growth and proliferation endpoints, such as primary and metastatic tumor burden, to document their efficiency as potential anticancer treatment modalities. The degree to which natural or engineered antitumor immunity can successfully target the highly invasive and migratory tumor cell subpopulation is poorly understood. As seen by the high recurrence rates in many solid malignancies, invasive/migratory tumor cells can evade the cytotoxic effects of chemotherapy, radiotherapy, and other treatments, as well as escape immunological detection and destruction (47, 73, 74). Cancer cell dissemination is regulated by a specialized network and subsets of myeloid cells, which form dedicated niches for the nurturing of migratory/invasive cancer cells (47, 50, 75–77). In this perspective, we propose that certain myeloid cell subsets, particularly perivascular M2-like macrophages, are contextually associated with cancer cell dissemination trajectories, offering a localized immunosuppressive niche to the metastasizing tumor cell population, while in the act of active dissemination. We conclude that thorough understanding of these immunosuppressive mechanisms in the tumor microenvironment at the molecular level will lead to more effective therapeutic targeting of cancer metastasis and will possibly improve the outcome of modern immunotherapies.

## The *Cancer Cell Dissemination Trajector*y: Tumor Microenvironment That Regulates the Initial Steps of the Metastatic Cascade

From earliest portrayals to more recent representations, two generic components have been distinguished as integral parts of the metastatic cascade, a cancer cell dissemination step and a cancer cell growth/proliferation step at the metastatic site, the latter also known as colonization step (78–84). Both these steps are regulated and may even be reinforced by a diverse array of biological programs in the tumor microenvironment, including epithelial-to-mesenchymal transition (EMT), invasion/migration, chemotaxis, and dormancy (85–92), among others. Recent advances in the underlying mechanisms of cancer cell dissemination have indicated that cancer cells that have undergone EMT, and thus have lost epithelial polarity and gained mesenchymal properties, participate in a reciprocal juxtacrine-paracrine signaling loop with tumor-associated macrophages (TAMs), eventually leading them to the underlying vasculature for subsequent intravasation. Cancer cell intravasation, however, does not occur along the entirety of the cancer-associated endothelium, but is rather restricted to specialized intravasation sites, known as Tumor MicroEnvironment of Metastasis (TMEM) doorways. In this section, we will briefly discuss the factors that underlie the spatial and functional relationship between the disseminating tumor cell subpopulation and the TMEM doorways, a critical ingredient that regulates the initial steps of the metastatic cascade in primary tumors.

Cancer cell intravasation doorways, also known as TMEM doorways, constitute intratumoral niches characterized by the physical juxtaposition of a tumor expressing high levels of the actin-regulatory protein Mammalian enabled (MENA), a perivascular macrophage and an endothelial cell, and represent an independent prognostic indicator of metastatic risk in breast cancer patients (93–96). Perivascular macrophages residing in TMEM doorways express the tyrosine kinase receptor TIE2, thus assuming an M2-like polarization status and tumor-promoting effects. Under the tight regulation of TIE2 signaling, TMEM macrophages secrete large amounts of vascular endothelial growth factor (VEGF), which in turn, functions in a paracrine fashion on the TMEM endothelial cell to promote the reversible breakdown of endothelial cell-to-cell adhesions, localized vasculature opening, and the subsequent intravasation of invasive/migratory tumor cells from the immediate area surrounding the TMEM doorway. Despite that the precise role of the TMEM tumor cell in the TMEM triad has not yet been clearly elucidated, high-resolution microscopy has suggested the presence of invadopodia stemming from TMEM tumor cells and extending in between the underlying vasculature (73). Thus, the current understanding is that TMEM tumor cells pinpoint the breaching point of the endothelial wall following TIE2-dependent TMEM doorway activation.

Formation of active TMEM doorways has not only been observed in primary tumors, but also in their respective loco-regional and distant metastatic sites, such as in the lymph nodes and lungs, respectively (75, 93, 97–99). Indeed, prior work has documented that established lymph node metastases attract TIE2^+^ macrophages in the perivascular niche, which in turn assemble TMEM doorways *de novo* (99). More importantly however, photoconversion experiments that can specifically label tumor cells in metastatic lymph nodes and observe their behavior in real time have indicated that cancer cells within metastatic foci are capable of utilizing TMEM doorways to re-disseminate to tertiary metastatic sites, such as to the lungs (99). Overall, these studies support TMEM doorway-mediated cancer cell dissemination as a universal mechanism of cancer cell dissemination at all stages of cancer progression.

Within the constantly evolving landscape of tumor cell heterogeneity, it is crucial to appreciate that not all tumor cells are equally capable of cancer cell dissemination *via* TMEM doorways. Rather, only a small subset of tumor cells in primary tumors is co-opted to utilize TMEM doorways for intravasation in the peripheral circulation. Expression profiling studies have specifically identified this subset as overexpressing an alternatively spliced isoform of the actin-regulatory protein Mammalian enabled (MENA), called MENA^INV^, and having concurrently lost expression of the antimetastatic and cell cohesion-promoting alternatively spliced isoform MENA11a (100–104). MENA is one of the key members of the Ena/VASP family of proteins, involved in regulation of cell movement, shape and adhesion (105), mainly through regulating actin filament polymerization and rate of filament elongation during the formation of cellular protrusions (106, 107). Cancer cells that overexpress MENA^INV^ are characterized by formation of extracellular matrix-degrading cellular protrusions, called invadopodia, by increased sensitivity to chemotactic factors in the tumor microenvironment such as epidermal growth factor (EGF) and hepatocyte growth factor (HGF), which both facilitate cancer cell invasion and migration (75, 98, 107–114). It is therefore not surprising that MENA^INV^-expressing tumor cells are preferentially co-opted for TMEM-mediated cancer cell intravasation. It should be noted that MENA^INV^-expressing cancer cells also share markers and phenotypic characteristics that indicate they have undergone epithelial-to-mesenchymal transition (EMT), which is a crucial landmark of metastatic dissemination (85, 115–118). However, in the current perspective we will primarily refer to the migratory/invasive tumor cell compartment as the MENA^INV+^ cancer cell subpopulation, given that prior studies have suggested that MENA isoform switching is crucial for the establishment of metastatic disease (103, 104, 106).

Multiphoton intravital imaging studies in live mice have suggested that MENA^INV^-expressing tumor cells migrate along collagen fibers with partnering TAMs in the tumor microenvironment. A well-described, reciprocal paracrine loop between the two cell types, involving colony stimulating factor-1 (CSF1) secretion from the tumor cell and epidermal growth factor (EGF) secretion from the macrophage, leads to the chemotactic attraction of one cell towards the other, coupling them in sequence in a unique migratory pattern called “cancer cell streaming” (104, 119, 120). The specific targeting of either of these factors, either pharmacologically or *via* genetic engineering, is sufficient by itself to disrupt cancer cell streaming and suppress cancer cell dissemination (104, 121–123). Eventually, MENA^INV+^ tumor cells reach down to the perivascular niche, whereby they utilize pre-existing TMEM doorways to intravasate into the blood vessel (104, 114, 124, 125). Chemotactic factors, such as hepatocyte growth factor (HGF) and stromal derived factor-1 (SDF1), either secreted by the cancer endothelium itself or by cells associated with the endothelium (e.g. TMEM macrophages), are responsible for the directed migration of the entire cancer cell “streams” towards the TMEM doorway (126, 127).

A few studies have previously investigated the mechanisms *via* which MENA^INV^ expression is induced in the migratory/invasive cancer cell subset. Although the exact mechanism has not been deciphered at the molecular level, there is strong indication that TAMs streaming with tumor cells are crucial for MENA^INV^ induction in the latter. Specifically, *in vitro* co-culture experiments have indicated an up to 50-fold increase in MENA^INV^ expression when tumor cells were co-cultured with macrophages, and this phenotype was demonstrated to be contact-dependent, suggesting that juxtacrine signaling loop may also be elicited during the cancer cell streaming behavior (124). In support to these observations, the *in vivo* depletion or suppression of macrophage differentiation has shown a significant reduction of cancer cell dissemination (128).

**Figure 1:**
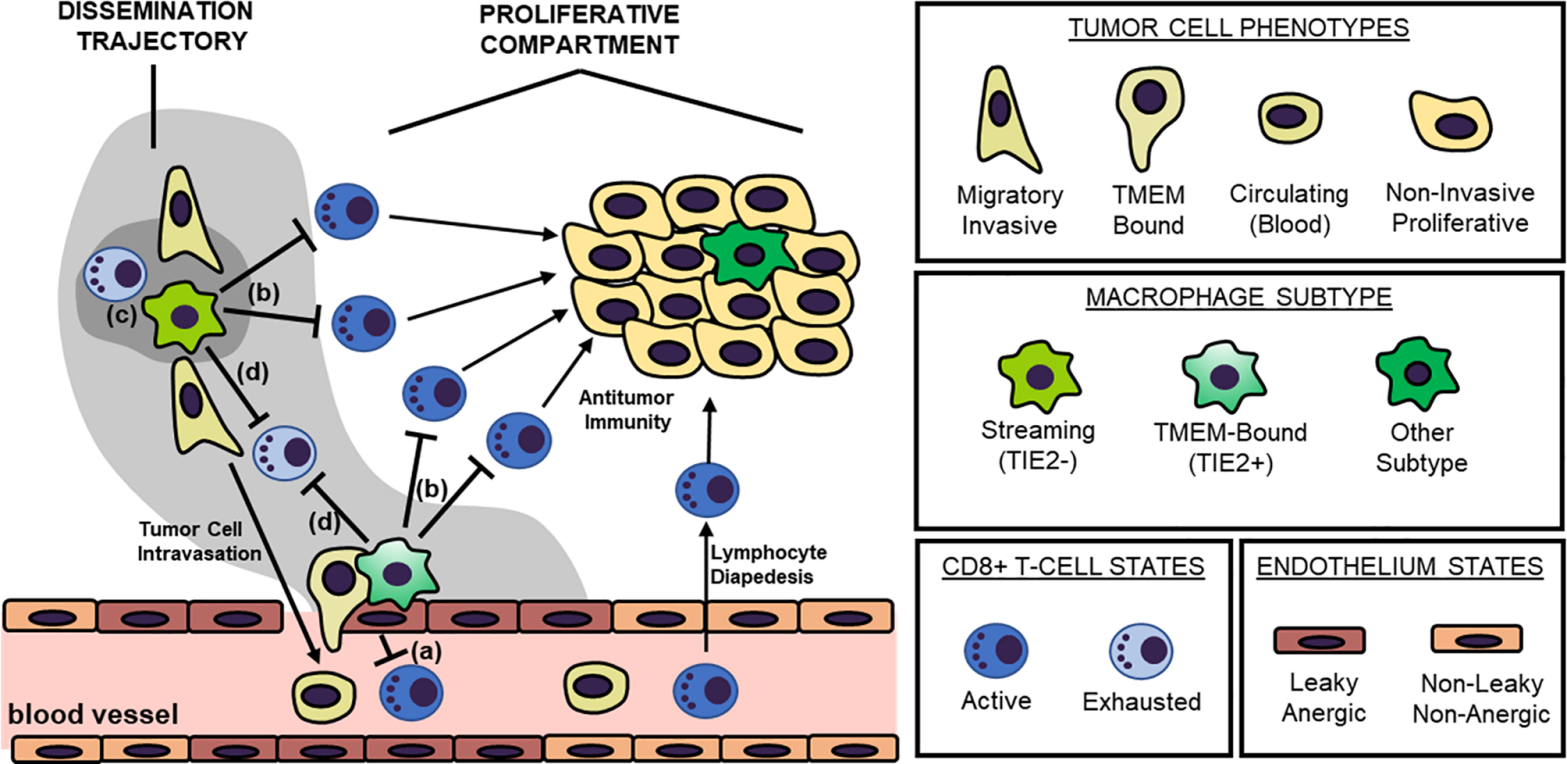
The “Dissemination Trajectory” Working Model of Metastatic Dissemination. Two major cellular prerequisites are necessary for cancer cell dissemination: a TMEM doorway and a highly invasive, highly migratory cancer cell subsets streaming toward TMEM doorways. TMEM doorways are composed of three cell types, a TIE2^+^ macrophage, an endothelial cell and a tumor cell forming an invadopod in the vasculature, and signaling conversation among these three cells results in localized vascular opening to facilitate transendothelial migration of the highly invasive, highly migratory cancer cell subset. The highly invasive and migratory cancer cell subsets participate in a reciprocal paracrine and juxtacrine signaling loop with intratumoral macrophages that do not express TIE2, resulting in the increased induction of the actin-regulatory protein MENA^INV^. Eventually, these interactions result in the so called “streaming migration”, which is directed toward TMEM doorways, and MENA^INV^-facilitated transendothelial migration and metastatic dissemination. TMEM doorways and their streaming MENA^INV+^ cancer cell subsets are herewith referred to as “dissemination trajectories”. These specialized microenvironments are distinguishable from other tumor compartments with rapidly dividing tumor cells that do not share similar molecular pathways, here described as “proliferative compartments”. Four layers of immunosuppressive mechanisms dominate within the dissemination trajectories, that result in the development of immune deserts further facilitating the process of metastatic dissemination. These mechanisms postulate that: (a) the TMEM endothelium is anergic, thus not allowing for T cell diapedesis; (b) dissemination trajectories do not support cytokine/chemokine matching for allowing T cell chemotaxis; (c) dissemination trajectories have a unique metabolic landscape that is refractory for T cell chemotaxis and/or function; and finally (d) dissemination trajectories are characterized by the induction of immune checkpoint signaling, that promoted exhaustion of T cells. Overall, tumor-associated macrophages (TAMs) within these dissemination trajectories play the pivotal role in regulating all four layers of immunosuppression, although secondary mechanisms have also been identified.

In this perspective, the described MENA^INV+^ migratory/invasive cancer cell population partnered with intratumoral TAMs during streaming, along with corresponding TMEM doorways used during the intravasation process, will be collectively referred to as the “*Dissemination Trajectory”* (**Figure 1**). Indeed, it is expected that different signaling pathways, cytokine/chemokine profiles, and metabolic patterns will characterize the dissemination trajectories versus the more proliferative compartments of solid tumors. Here, we explore dissemination trajectories as immunosuppressive landscapes, in an effort to explain current translational and clinical observations on why natural or engineered antitumor immunity is not efficient in preventing the metastatic cascade, albeit demonstrating promising results in eliminating tumor growth potential.

## The Cancer Cell Dissemination Trajectory as an Immunosuppressive Niche

**Figure 2:**
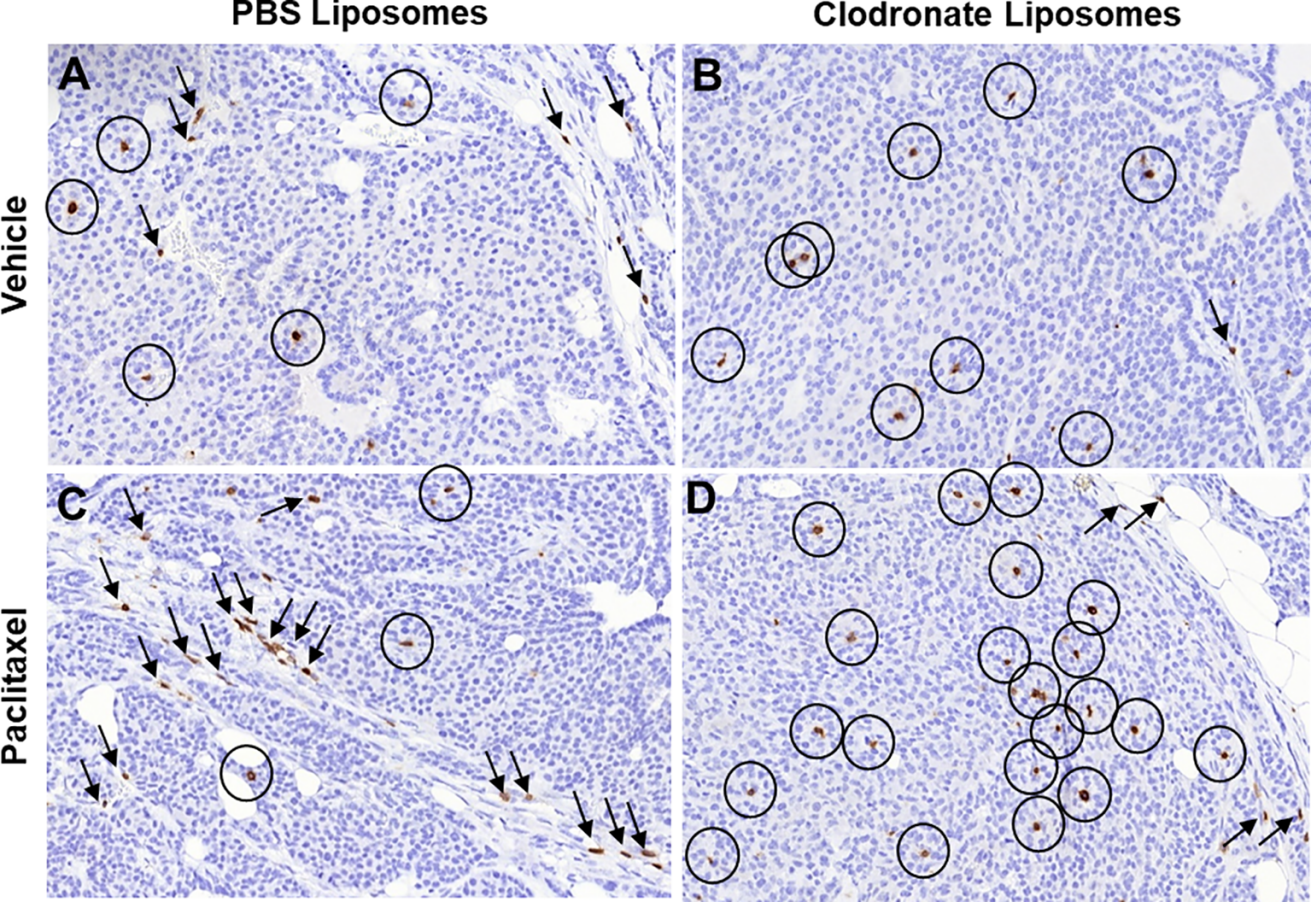
Immunohistochemical indication of how different pharmacologic modifications of the immunosuppressive tumor microenvironment may affect T cell trafficking into tumors. **(A–D)** Immunohistochemistry for T cell specific marker CD3 in tumor sections from mouse mammary tumor virus – polyoma middle T antigen (MMTV-PyMT) mice, developing spontaneous breast carcinomas. The images are high power fields (x40), representative from a total of three mice in each experimental condition. Circles, CD3^+^ T cells infiltrating the tumor nests; Arrows, CD3^+^ T cells infiltrating the tumor stroma. Notice the significant changes in intratumoral versus stromal T cell infiltration upon different treatments that modify the immunosuppressive microenvironment.) In breast carcinoma, T cells are found in both tumor cell nests and the tumor stroma **(A)**. Upon macrophage depletion with clodronate liposomes, most T cells can leave the stroma and penetrate the tumor cell nests **(B)**. However, treatment with cytotoxic chemotherapy is known to induce lymphocyte infiltration and significantly larger number of T cells is found compared to the vehicle **(C)**. Notably however, most of these T cells are restricted in the tumor stroma, as chemotherapy attracts immunosuppressive myeloid cells at the same time, resulting in lymphocyte exclusion **(C)**. If such immunosuppressive myeloid cells are depleted through clodronate liposomes in chemotherapy-treated tumors, the increased influx of T cells is now relocated in the tumor nests **(D)**. *Immunohistochemistry was performed in archival tissue from experiments originally conducted in the manuscript by Karagiannis et al*. (139)*, in which ethical approval for the use of the experimental mice was also obtained* (139).

A substantial amount of preclinical and clinical studies has indicated that tumor-associated myeloid cells, predominantly tumor-associated macrophages (TAMs), neutrophils, and myeloid-derived suppressor cells (MDSCs), sustain an immunosuppressive tumor microenvironment, which is particularly refractory to both T cell trafficking and antitumor T cell functions (4, 129–134). Extensive research in this field has additionally concluded that the specific targeting and/or elimination of this myeloid-driven immunosuppressive program can render the natural, induced, and engineered immunological responses against tumors more concrete and effective (135–137). In line with the above, here we first provide proof-of-principle evidence of this notion using the Mouse Mammary Tumor Virus Polyoma Middle-T antigen (MMTV-PyMT) mouse model of breast carcinoma, which successfully recapitulates human breast cancer progression (138). During the natural progression of MMTV-PyMT carcinomas, T cells are spatially restricted to the peritumoral stromal sheaths and are visually excluded from multicellular tumor cell cohorts (**Figure 2A**), insinuating structural and/or functional impediments of intratumoral T cell trafficking. However, upon the pharmacological depletion of TAMs *via* the administration of clodronate liposomes, intratumoral T cell trafficking is clearly improved (**Figure 2B**), pinpointing TAMs as the responsible structural and functional impediments to T cell trafficking. It has been previously shown that immune cells can excessively infiltrate primary tumors as a result of a cytokine surge, induced by cytotoxic factors, such as chemotherapy treatment (47, 73, 74, 139). Indeed, administration of paclitaxel, a taxane-based chemotherapy known to inflict prometastatic modifications as a consequence of a cytokine surge (139–144), results in a dramatic increase of TILs, which are otherwise restricted to the peritumoral stromal sheaths (**Figure 2C**). This distribution pattern appears to be the consequence of immunosuppressive TAMs, because clodronate-mediated depletion of TAMs in the chemotherapy setting facilitates the intratumoral trafficking of T cells that have responded to the chemotherapy-driven cytokine surge (**Figure 2D**). Of note, similar observations by other groups have corroborated our findings using a diverse array of macrophage suppression or re-polarization strategies (77, 145, 146). In conclusion, these experimental data along with accompanied literature collectively demarcate the detrimental impact of TAMs in T cell trafficking and distribution in primary tumors.

As described above, specialized tumor microenvironments within primary carcinomas comprising of TMEM doorways and their associated prometastatic MENA^INV+^ cancer cell compartments, herewith defined as *dissemination trajectories*, are both structurally and functionally supported by distinct TAM subsets (47). Given the experimental and literature evidence on the immunosuppressive properties of TAMs described above, here, we surmise that dissemination trajectories signify immunosuppressive niches, reminiscent of *immune deserts*. The term “immune dessert” is used here as an interchangeable term for collectively describing tumor microenvironments with immune excluded and immune desert phenotypes, as defined in multiple prior studies (147–150). It is hereby suggested that at least four distinct mechanisms may contribute to the function of dissemination trajectories as immune deserts in the primary tumor microenvironment: First, dissemination trajectories are sites of endothelial anergy; Second, they represent sites of lymphocyte exclusion; Third, they represent sites of metabolic reprograming, refractory to anti-tumor lymphocyte functions; Fourth, they constitute sites of lymphocyte exhaustion. Collectively, the aforementioned immunosuppressive mechanisms (**Figure 1**) significantly undermine the capacity of the tumor-infiltrating lymphocytes for targeting the disseminating cancer cell population, thus allowing for a narrow, but solid window of opportunity for the successful execution of the initial steps of the metastatic cascade.

### (a) Dissemination Trajectories as Beacons of Endothelial Anergy

Lymphocyte migration needs to be precisely coordinated to contribute to effective T cell trafficking in both physiological and neoplastic contexts. This process can be summarized into selectin-dependent leukocyte rolling, chemokine-driven integrin activation, integrin-dependent leukocyte tethering in the vascular wall, and leukocyte diapedesis (8, 9, 151–153). It should be mentioned that this process is primarily mediated by lymphocyte-endothelial cell interactions, and as a consequence, the integrity and functionality of the endothelium in either a physiological or neoplastic context, could have a dramatic effect on T cell trafficking. Under the control of growth factors and abnormal contextual signals, the tumor (neo)vasculature often displays a high angiogenic potential coupled to irregular distribution, enlarged vessels, excessive branching morphology, microhemorrhaging, and disturbed blood flow, when compared to traditional blood vessel architecture and physiology (154–158). Another decisive factor contributing to tumor endothelium instability, and consequently to defective T cell trafficking into the tumor tissue, is the failure to support endothelial integrity and functions *via* adequate mural cell (e.g. pericyte) coverage (159–162). One could intuitively, but erroneously, assume that high endothelium instability/permeability should render immune cell trafficking much easier. However, circulating lymphocytes require specialized molecular signatures (e.g. selectin, integrin and chemokine profiles) in tissue endothelial barriers to help with their homing into tissues (163–167). These molecular signatures, which are magnanimously present in High Endothelial Venules (HEVs) of various lymphoid organs for example (168–171), are characteristically disrupted or absent in tumor endothelia, rendering them “leakier” and insensitive to pro-inflammatory signals (172). This phenotype, known as “endothelial anergy”, is characterized by impaired adherence of effector T cells to the endothelial cells and their subsequent extravasation to the tumor microenvironment (9, 173).

Although macrophages provide essential trophic factors to facilitate generation and retention of pericytes in certain developmental contexts (174, 175), at least one study has previously indicated that TMEM doorways are devoid of NG1^+^ pericyte coverage (128), signifying one potential signature of endothelial anergy at TMEM doorways. In yet other studies, it has been shown that M2-polarized TAMs may in contrast support macrophage-pericyte interactions in the tumor microenvironment, but such interactions lead to enhanced neovascularization and tumor progression (176–179), again flagging the immediate surroundings of M2-like TAMs as potential niches of endothelial anergy.

For a long time, it has been theorized that the tumor vasculature is under constant and simultaneous control of proangiogenic and antiangiogenic factors, with vascular endothelial growth factor-A (VEGFA) representing a well-known paradigm of angiogenesis inducers (155, 180–186). However, prior evidence also suggests that different blood vessel subtypes in the tumor microenvironment do not all respond homogeneously to anti-VEGF treatment (187), inferring the presence of contextual factors promoting heterogeneity in VEGFA expression and activity. Indeed, the increase of VEGF around TMEM doorways may be the cause of the overall heterogeneity of VEGF expression around blood vessels in tumors. Under the transcriptional control of the Ang2-Tie2 signaling axis, TIE2^+^ TMEM macrophages can locally release large quantities of proangiogenic factors, most prominently VEGFA, which is critical for both eliciting an angiogenesis program and sustaining TMEM function and TMEM-mediated cancer cell dissemination (128, 188–190). Importantly, VEGFA regulates blood vessel wall permeability *via* a variety of mechanisms, for instance *via* increasing endothelial cell fenestration at lower concentrations, or *via* breaking down and dissolving the endothelial cell adherens and tight junctions at higher concentrations (191–197). The latter is especially critical in the process of metastasis because it provides an effective paracellular passageway for the disseminating cancer cell subpopulation into the blood circulation (128). Indeed, the conditional ablation of the VEGFA gene *via* targeted expression of Cre recombinase under the control of the macrophage-specific promoter that regulates transcription of the colony stimulated factor-1 receptor (CSF1R) in a mouse model of breast carcinoma results in successful assembly of TMEM-doorways, which are otherwise entirely incapable of breaking down endothelial junctions and facilitating cancer cell transendothelial migration and intravasation (128). Overall, these data suggest that TMEM doorways within the dissemination trajectories reflect to TMEs with high VEGFA expression and activity, suggesting that they function as candidate beacons of endothelial anergy within the tumor microenvironment.

TMEM doorways are functionally regulated by M2-like TAMs, which represent a prominent source of angiogenic molecules in the perivascular niche (47, 51, 75, 189, 190, 198–201). Besides the well documented VEGFA, TAMs release a plethora of other proangiogenic factors, such as tumor necrosis factor-α, basic fibroblast growth factor, thymidine phosphorylase, urokinase-type plasminogen activator, adrenomedullin, and semaphorin-4D (47, 189, 190, 201–205). These proangiogenic factors are known to downregulate the expression of adhesion molecules (ICAMs, VCAMs, and selectins), which are actively involved in lymphocyte trafficking, thus resulting in endothelial anergy and lymphocyte tolerance (9, 172).

**Figure 3:**
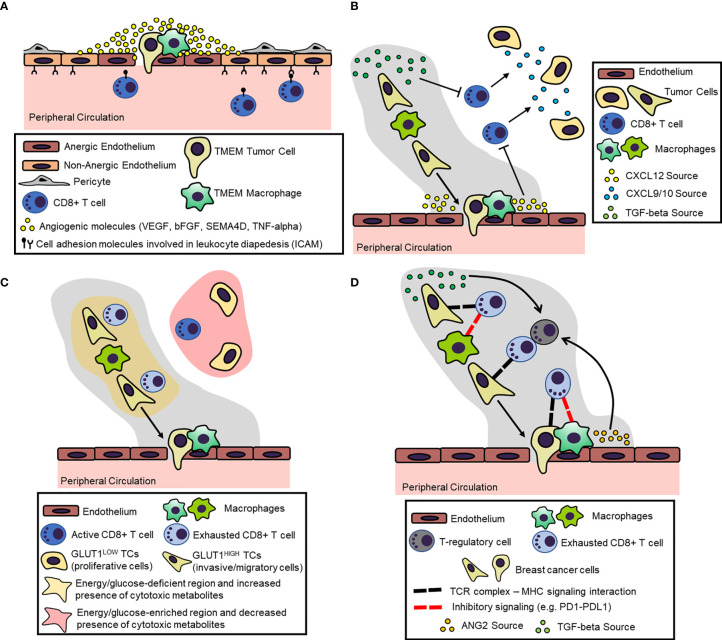
Proposed Mechanisms for the Induction and Maintenance of an Immunosuppressive Microenvironment within the Dissemination Trajectory. **(A)** Dissemination trajectories as beacons of endothelial anergy. Perivascular (TMEM doorway) macrophages secrete a number of proangiogenic factors (e.g. VEGFA) in the peri-TMEM area, which downregulate cell adhesion molecules in endothelial cells critical for lymphocyte diapedesis, thus resulting in “locally” anergic endothelium. **(B)** Dissemination trajectories as crossroads for T cell exclusion. Cytokine/cytokine receptor mismatching mechanisms within the dissemination trajectories result in the exclusion of T cells. For example, prometastatic macrophages suppress the expression CXCL9/10 within the dissemination trajectories, which function as the primary chemoattractants for T cells. Instead, dissemination trajectories are characterized by the expression of other cytokines/chemokines, like TGF-beta and CXCL12, which act as repellents for T cells. **(C)** Dissemination trajectories as primers for metabolic burdening of T cells. Highly migratory tumor cells within the dissemination trajectories tend to upregulate glucose transporters (e.g., GLUT1), which on one hand reduces the bioavailable energy resources (i.e., glucose), while on the other hand, may produce metabolites. This metabolic landscape is burdensome for immune cells, resulting in T cell exclusion and exhaustion. **(D)** Dissemination trajectories as checkpoints for T cell exhaustion. Chronic TCR signaling within the dissemination trajectory along with overexpression of inhibitory ligands (e.g., PDL1) by the prometastatic macrophages may result in T-regulatory (Treg) cell expansion and CD8+ T cell inactivation/exhaustion.

In summary (**Figure 3A**), TMEM doorways likely serve as proponent components of endothelial anergy in the tumor microenvironment, subduing intratumoral recruitment of CD8^+^ T cells. Possible contributors of endothelial anergy at TMEM doorways are the reported defects in pericyte coverage, as well as the localized, high concentration of VEGFA and other proangiogenic molecules secreted by the TMEM macrophage. These mechanisms may together prompt a highly permeable vasculature at TMEM doorways, which is otherwise unable to support T cell trafficking due to the lack of characteristic molecular signatures for lymphocyte diapedesis.

### (b) Dissemination Trajectories as Crossroads for Lymphocyte Exclusion

Among the critical mechanisms leading to inadequate T cell trafficking into solid tumors are those culminating in mismatching between bioavailable chemokines in the tumor microenvironment and chemokine receptors expressed on the surface of cytotoxic T cells (206). The disruption of the immunosuppressive chemokine/cytokine network either pharmacologically or *via* genetic manipulations in animal models can therefore reliably covert the tumor microenvironment into a receptive niche for T cell trafficking and further sensitize tumors to immunotherapy (207). The dissemination trajectories are functionally and contextually associated with distinct macrophage subtypes, which represent a prominent source of immunosuppressive cytokines and chemokines in the tumor microenvironment (47, 73–75, 206). As mentioned, perivascular TMEM doorway macrophages, express high levels of the tyrosine kinase receptor TIE2 (also known as CD202b), and the mannose receptor MRC1 (also known as CD206), suggesting that they are skewed towards an M2 (or M2-like) phenotype according to the traditional macrophage polarization spectrum (47, 119, 208, 209). In this perspective, we support the working model that M2-like macrophages within dissemination trajectories represent the major orchestrators of chemokine/chemokine receptor mismatching that leads to inadequate CD8^+^ T cell trafficking (206).

Peripheral monocytes are usually recruited within tumors *via* the CCL2/CCR2 chemokine pathway and transdifferentiate into M2-like macrophages under the regulation of the CSF1/CSF1R pathway (210). It is now strongly documented that CSF1-dependent macrophage polarization into M2-like phenotype leads to the acquisition of an immunosuppressive macrophage subtype, characterized by T cell exclusion (145, 206, 211–213). Indeed, the depletion of tumor-associated macrophages *via* inhibiting either CSF1/CSF1R or CCL2/CCR2, are both capable of overcoming T cell exclusion within tumors (145, 214). There is sufficient evidence that M2-like macrophage functions are antagonistic to Th1 immunological responses, which would theoretically favor antitumoral immunity. Specifically, M2-like macrophages may suppress the interferon-gamma (IFN)-mediated responses that culminate in the induction of CXCL9 and CXCL10 chemokines, which, in turn, are able to attract CXCR3^+^CD8^+^ memory T cells (215). The critical association between CXCR3-binding ligands CXCL9/10 and CD8^+^ T cell trafficking has been well documented (216–220). Although the dominance of M2-like macrophages within the dissemination trajectories can by itself account for the suppression of such favorable Th1 immunological responses, several macrophage-independent mechanisms of Th1 suppression have also been reported in this context. For example, certain tumors (e.g. ovarian carcinomas) can use epigenetic mechanisms to silence the expression of CXCL9 and CXCL10. Moreover, nitrosylation by reactive oxygen species (ROS) in the tumor microenvironment may result in altered proteolytic processing of CXCL11, another chemoattractant of CD8+ T cells (221), which incapacitates its binding-induced signaling (222).

It has been demonstrated that once homed in tumors under the control of CCL2/CCR2 and CSF1/CSF1R pathways, M2-like macrophages begin to also express the chemokine receptor CXCR4, possibly under the control of the pleiotropic cytokine TGFβ (210). The *de novo* expression of CXCR4 may force prometastatic macrophages into a unidirectional migration toward the perivascular niche where CXCL12, the chemokine ligand of CXCR4 is abundantly expressed, and where they eventually assemble TMEM doorways (210). Although many sources of TGFβ within the tumor microenvironment have been reported (223–225), human monocytes and macrophages can also activate TGFβ *via* the expression of integrin αvβ (226). TGFβ has been previously documented as among the strongest immunosuppressive cytokines, capable of excluding T cells from human and murine tumors (227, 228). These observations collectively suggest that TGFβ expression within dissemination trajectories represent a critical mechanism of lymphocyte exclusion as a result of cytokine/cytokine receptor mismatching.

As mentioned, CXCR4^+^ macrophages within dissemination trajectories can chemotactically respond to the presence of the CXCL12 ligand at the perivascular niche (210). Prior evidence suggests that mesenchymal stromal cells, such as cancer-associated fibroblasts (CAFs) and possibly mural cells coating the blood vasculature, serve as the primary source of CXCL12 production and secretion (229–234). In a recent model of cancer cell dissemination, the chemotactic migration of CXCR4^+^ macrophages with their partnering MENA^INV+^ tumor cells, has been rendered as the possible driving force for the observed streaming migratory behavior within dissemination trajectories (210). Moreover, a concrete body of evidence supports that intratumoral distribution of CXCL12 inversely correlates with the presence of T cells (235), although it is not yet clear whether the CXCL12/CXCR4 pathway can directly suppress T cell trafficking into CXCL12-enriched microenvironments (230, 236). Indeed, pharmacological inhibition of the CXCL12/CXCR4 pathway alleviates the tumor microenvironment from the lymphocyte exclusion phenotype (236). On one hand, CXCL12 appears to be a critical chemokine for cancer cell dissemination (231, 233, 237, 238); still, it may comprise a major chemokine/chemokine receptor mismatching mechanism for the trafficking of T cells into dissemination trajectories.

In summary (**Figure 3B**), the immunosuppressive M2-like macrophages may orchestrate the expansion of a cytokine/chemokine network, which excludes T cells from the dissemination trajectories. Foremost, M2-like macrophages seem to directly suppress the expression of the CXCR3-binding ligands CXCL9 and CXCL10, which are the primary chemokine attractants for CD8^+^ T cells. Moreover, the reciprocal interactions among disparate cells within dissemination trajectories seem to be highly dependent on the induction and contextual expression of several cytokines and chemokines, including but not limited to CXCL12 and TGFβ, which may disrupt lymphocyte trafficking and exclude CD8^+^ T cells from the landscape.

### (c) Dissemination Trajectories as Primers for Metabolic Burdening of Lymphocytes

In general, sugars, amino acids, and fatty acids are the major fuel sources utilized by eukaryotic cells, but rapidly proliferating tumor cells tend to exhaust them, thus subjecting both tumor and immune cells to nutrient-deficient microenvironments and imposing considerable bioenergetic constraints on their functions (76, 151, 239–242). Cancer cells tend to upregulate the expression of glucose transporters, such as GLUT1 (243, 244), amino acid transporters, such as ASCT2 and LAT1 (245–247), and fatty acid elongation enzymes, such as FAS (248–250), to facilitate their adaptation to energy-deficient microenvironments. This metabolic reprogramming does not only limit nutrient availability for cytotoxic CD8^+^ T cells, but may also generate metabolic byproducts that may overwhelm T cell function, survival, and expansion (151). In this chapter, we briefly explore certain mechanisms, *via* which the metabolic landscape within dissemination trajectories may interfere with lymphocyte trafficking and function.

Foremost, the metabolic machinery of MENA^INV+^ tumor cells within dissemination trajectories remains poorly understood. However, it is generally known that fatty acids are primarily required by rapidly dividing tumor cells to form new plasma membrane lipid bilayers, thus explaining why most tumors overexpress FAS and malignant transformation depends on lipogenesis (251). However, neither migratory tumor cells nor effector T cells seem to heavily depend on fatty acid oxidation, although the development of antitumor memory T cells is affected (252–254), suggesting that such pathways may not be as immunocompromising within the dissemination trajectories. On the other hand, there are certain lines of evidence suggesting that the highly migratory/invasive cells that have undergone EMT tend to express high levels of the glucose transporter GLUT1, which partially supports high energy demands for the active process of invasion and migration (255, 256). Accordingly, it has been shown that proteolytic modifications of the extracellular matrix by highly migratory cells *per se* can also promote GLUT1 expression and aerobic glycolysis (257). Concomitantly, GLUT1 overexpression has been associated with low T cell trafficking in renal cell and squamous cell carcinomas (258, 259), suggesting that dissemination trajectories could potentially limit both T cell trafficking and their functional capacity in a GLUT1-dependent manner.

The metabolic landscape within dissemination trajectories may also impair T cell functions through generation of immunosuppressive metabolites and byproducts, not only *via* the direct competition for energy resource availability. For example, indoleamine 2,3-dioxygenase (IDO), an enzyme that converts tryptophan into kyunerines (260), is a well-established suppressor of CD8^+^ T cell infiltration into tumors and most of the associated antitumor T cell responses (132, 261). In addition, diminished tryptophan deposits in IDO^High^ tumor microenvironments can prevent T cell proliferation, while kyunerines can promote T cell death and interference with TCR signaling (132, 261, 262). Despite that dendritic cells have been identified as major inducers of IDO within the immune microenvironment (261), TAMs can also participate in IDO-mediated tryptophan metabolism under certain contexts (263–265), suggesting that dissemination trajectories may be characterized by the accumulation of immunosuppressive metabolites.

In summary (**Figure 3C**), dissemination trajectories are associated with a metabolic landscape that results in diminished T cell trafficking into tumors and associated antitumor T cell functions. On one side, highly migratory tumor cells within the dissemination trajectories may successfully outcompete TILs for the scant availability of energy resources, such as glucose, because they tend to upregulate corresponding transporters (e.g., GLUT1). On the other hand, TAMs within the dissemination trajectories may be engaged in metabolic pathways that not only deplete essential elements (e.g., tryptophan), but also produce immunosuppressive metabolites along the process (e.g., IDO-induced kyunerines).

### (d) Dissemination Trajectories as Checkpoints for Lymphocyte Exhaustion

In recent years, it has been suggested that effector T cells (CD4^+^ and CD8^+^), which infiltrate tumors tend to exhibit impaired functional and proliferating capacity, characterized by progressive loss of their ability to produce their characteristic effector cytokines (i.e., TNF-α, IFN-γ, IL-2) and lyse tumor cells, a state described as *lymphocyte exhaustion* (45, 151, 207, 266–269). The existence of this particular phenotype is further corroborated through experimental evidence showing that certain cancer immunotherapies, such as those that specifically target immune checkpoint pathways, may alleviate T cell exhaustion, and restore the ability to eradicate cancer cells (270). In this section, we propose that dissemination trajectories rich in M2-like immunosuppressive macrophages can yield a contextual milieu that promotes T cell exhaustion, potentially accounting for the lack of treatment response seen in many patients following checkpoint therapies.

Similar to the case of chronic viral infections, the most prominent hallmark of T cell exhaustion in the tumor microenvironment is the co-expression of a wide range of immune checkpoint receptors by the T cells (271, 272). These inhibitory receptors primarily include programmed cell death protein 1 (PD1), lymphocyte activation gene 3 protein (LAG3), T-cell immunoglobulin domain and mucin domain protein 3 (TIM3), cytotoxic T lymphocyte antigen-4 (CTLA4), band T lymphocyte attenuator (BTLA) and T-cell immunoglobulin and immunoreceptor tyrosine-based inhibitory motif domain (TIGIT) (273). Although it is beyond the scope of the current perspective to delineate the detailed biology of these immune checkpoint pathways, it should be mentioned that intracellular signaling *via* these receptors in T cells can generally lead to functional deficiencies characteristic of the lymphocyte exhaustion phenotype (274–279). However, in a certain context, PD1^+^TIM3^+^ tumor-infiltrating T cells were functional despite the co-expression of both immune checkpoint receptors, suggesting that certain competitive intracellular pathways to unruly T cell exhaustion may also exist (280). It has been generally known that TAMs are prominent inducers of T cell exhaustion in the tumor microenvironment through interference with immune checkpoint control. For example, TAMs from renal cell carcinoma patients induce the skewing of autologous blood derived CD4^+^ T cells towards an exhausted phenotype, with decreased production of effector cytokines and enhanced expression of PD1 and TIM3 (281). Of all immune checkpoint pathways mentioned above, the prominent expression of PDL1, a ligand for PD1, and B7-H4, a ligand for CTLA4, are perhaps the most well-known immunosuppressive mechanisms leading to macrophage-driven T cell exhaustion (282–286). Of particular interest is the fact that ligands for immune checkpoint receptors are mostly expressed by M2-like macrophages, which are also integral components of TMEM doorways, providing another attractive theory for immune evasion by the migratory/invasive cancer cell subpopulation within the dissemination trajectories.

Prior research has suggested that chronic T cell receptor (TCR) signaling in functional T cells can normally lead to elevated expression of inhibitory receptors, such as PD1, TIGIT and CTLA4 (271, 287). This observation further postulates that increased expression of these inhibitory receptors in TILs may accordingly be the result of chronic exposure to neoantigens and/or persisting tumor antigens (151, 268). However, the expression of inhibitory receptors in TILs is markedly higher compared to those in functional T cell states, suggesting that other factors, possibly microenvironmental ones, may be responsible for increased immune checkpoint control and lymphocyte exhaustion (151). In accordance with these observations, prior experimental evidence has demonstrated that certain cytokines, often expressed in the tumor microenvironment (tumor cells, cancer-associated fibroblasts, immune cells, adipocytes), such as angiopoietin-2 (ANG2), interleukin-10 (IL10), and transforming growth factor-β (TGFβ), are sufficient for T cell exhaustion and suppression of anticancer immunity (288–291). Although, this cytokine network leads to lymphocyte exhaustion through a variety of mechanistic pathways, both direct and indirect *via* the expansion of CD4^+^CD25^high^FOXP3^+^ T-regulatory (Treg) cells, have been suggested (270, 272, 273, 292). Certain of these cytokines, especially TGFβ, have been discussed in prior chapters with regards to their functional relevance within dissemination trajectories. Others, like ANG2, are also critical for cancer cell dissemination, as ANG2-dependent activation of TIE2 receptor in the TMEM macrophage leads to the localized production and secretion of VEGF, which in turn, is critical for TMEM-associated vascular opening and the transendothelial migration of MENA^INV+^ tumor cells (75, 98). Therefore, it seems that dissemination trajectories are enriched in cytokines that not only promote lymphocyte exclusion, but also lymphocyte exhaustion.

In summary (**Figure 3D**), T cells that are not excluded from and manage to eventually infiltrate dissemination trajectories have acquired an “exhausted” phenotype rendering them unable to produce effector cytokines and successfully target tumor cells. This phenotype is regulated by an abnormally high expression of immune checkpoint receptors, such as PD1, CTLA4 and TIM3, at their surface. Among other cells, M2-like immunosuppressive TAMs within dissemination trajectories express a spectrum of corresponding ligands for these inhibitory receptors, thus offering immunosuppressive “sanctuaries” around the exhausted CD8^+^ T cells. Furthermore, the cytokine network within the dissemination trajectory, including primarily TGFβ and ANG2, among other factors, serves as a critical driver of Treg expansion and inhibitory receptor overexpression, thus maintaining and perpetuating the dysfunctional T cell states.

## Conclusions and Future Perspectives

In recent years, the molecular/cellular investigation of the immune tumor microenvironment and the comprehensive studying of the immunosuppressive mechanisms harbored therein have been at the frontier of cancer research, as an attempt to improve the already promising landscape of cancer immunotherapy (1, 86, 132, 135, 151). In this regard, we offer a fresh perspective on the topic by distinguishing disparate sets of immunosuppressive mechanisms in different tumor microenvironments. In particular, here we focused on analyzing multiple layers of immunosuppression, which involve mechanisms preventing T cell trafficking and mechanisms promoting T cell exhaustion within the specialized microenvironments dedicated to cancer cell dissemination (i.e., dissemination trajectories). This unique distinction serves a dual purpose: First, it offers an attractive explanation on why most immunotherapies do not target the migratory/invasive tumor cell subpopulation but instead are primarily restricted in promoting antitumor immunity within the more proliferative - less migratory tumor compartment. Second, it provides a rational framework on thinking the diverse immunosuppressive mechanisms as a multilayered obstacle against antitumor immunity, clearly suggesting that we should focus on targeting the immunosuppressive “network” rather than a “pathway” to be able to either restore the natural or orchestrate an engineered antitumor immunity. This perspective certainly does not aim at understating the importance of studying mechanisms of T cell exclusion and exhaustion in the proliferative and/or the cancer stem cell niches of the tumor microenvironment, given that targeting these microenvironments is also critical for establishing efficient anticancer immunity. However, this perspective aims at drawing significant attention to the frequently neglected concept of cancer cell dissemination, which may lead to a significant burden of dormant tumor cells in the distant metastatic sites, which may eventually grow into overt once they have found a way to avoid immunological detection and acquired resistance to immunotherapy or other therapeutic modalities (293). Therefore, the rational targeting of immunosuppressive mechanisms within the dissemination trajectories would serve as a promising *antimetastatic therapy*, given that its purpose would be to improve T cell trafficking and to alleviate T cell exhaustion, thus rallying an immunological attack against the migratory/invasive cancer cell population while in the act of departure from the primary tumor.

In pursuit of understanding the escape of migratory/invasive (MENA^INV+^) cells from antitumor immunity, here, we propose a unified model with at least four distinct layers of immunosuppression. Foremost, we propose that endothelial anergy and cytokine/cytokine receptor mismatching mechanisms do not allow for robust T cell trafficking within dissemination trajectories, and, in case that these mechanisms are somehow breached, alternative mechanisms promoting T cell exhaustion from either metabolic burdening or immune checkpoint control may become dominant (**Figure 1**). It should be noted that all these individual mechanisms are strictly context-dependent and may occur simultaneously within dissemination trajectories, not in tandem. As a consequence, therapeutic targeting of these mechanisms for purposes of improving cancer chemotherapy and/or immunotherapy should consider all the aforementioned categories of immunosuppression, because counteracting a single one would likely be inadequate. Fortunately, therapeutic strategies that target each individual immunosuppressive layer in our model (**Figure 1**) are in development. Therefore, the greatest challenge for the next decade will fall back to eliciting the most appropriate combinations to successfully cripple the immunosuppressive niche within the tumor microenvironment, including within the dissemination trajectory. For example, prior reported antiangiogenic approaches aimed at promoting blood vessel normalization were shown to concurrently disrupt endothelial anergy, resulting in (re)sensitizing tumor blood vessels to lymphocyte diapedesis and improved T cell trafficking (294). Furthermore, immune checkpoint receptor/ligand blockade (primarily of CTLA4, PD1, and PDL1) with monoclonal antibodies has emerged as a successful therapy against intratumoral T cell exhaustion in human patients (1, 69, 291, 295, 296). Combining such antiangiogenic therapies with immune checkpoint blockade could represent the most attractive strategy to counteract immunosuppression and render cancer immunotherapy more successful (297).

Most conclusions regarding the immunosuppressive cues described in this review article have risen from literature evidence on the immunosuppressive properties of M2-like macrophages in general. The most critical aspect of the working model of spatial immunosuppression (**Figure 3**) is the contextual positioning of M2-like TAMs within the dissemination trajectories, either those represent “streaming” or “TMEM-doorway” macrophages. Therefore, the immunological properties of all the distinct tumor compartments are attributed to the topographical enrichment of M2-like macrophages within the dissemination trajectories rather than to unique or specific M2-like macrophage phenotypes.

As mentioned earlier, TMEM doorways are also formed in diverse metastatic sites, such as in the lungs and lymph nodes, and such *de novo* dissemination machineries may participate in the re-dissemination of cancer cells to tertiary sites, accelerating metastatic burden (97, 99). Indeed, analysis of TMEM doorways in secondary/metastatic sites suggests that their ensuing biology can mimic to great extent the biological programming of cancer cell dissemination observed in primary tumors (97, 99). It would therefore be interesting to investigate in the future if identical or similar immunosuppressive cues are recapitulated in the metastatic microenvironments that assemble “re-dissemination machineries”.

The deeper we delve into the complex circuitries involving immune cells and their associated cytokine/chemokine signatures in the tumor microenvironment, the necessity for more sophisticated technologies to study the processes they are involved with, will constantly emerge. Indeed, conclusions from many studies included in this perspective would be impossible to be drawn in the absence of high-throughput technologies for multiplex imaging and/or single cell expression profiling. In addition, high-resolution imaging (e.g., intravital fluorescence microscopy and planar bioluminescence imaging) has yielded important spatiotemporal data at single cell resolution, furthering our understanding on the immunological pathways supporting the active process of cancer cell dissemination (298–300). To complement the aforementioned efforts, such emerging technologies will additionally provide feasible tools for analyzing mutation antigen profiles, gene signatures and epigenetic modifications of both tumor and immune cells, the breadth of antibody responses, as well as the magnitude, homing capacity, cytotoxic function, and T cell receptor (TCR) repertoires of tumor-infiltrating lymphocytes. Overall, we anticipate that new technologies in this intriguing field of research will bring us a step closer to achieving personalized medicine and more promising immunotherapies.

In brief, here we describe an alternative perspective that tumor microenvironments dedicated to cancer cell dissemination may elicit strong immunosuppressive cues that prevent T cell trafficking and promote T cell exhaustion, processes that undeniably facilitate the initial steps of the metastatic cascade. Interestingly, these mechanisms are primarily orchestrated by certain well-recognized subsets of tumor-promoting TAMs (e.g., TIE2^+^ TAMs), and their corresponding cytokine/chemokine network deployed around the cancer cell dissemination machinery. This working model of compartmentalized “immunosubversion” provides the groundwork for future studies on alleviating the immunosuppressive milieu for more optimal cancer immunotherapies.

## Author Contributions

SA, GKi, PF, DE, DM, MO, and GKa: wrote the manuscript. LS and GKa: produced data and designed **Figure 2**. GKa: designed illustrations for **Figures 1** and **3**. All authors contributed to the article and approved the submitted version.

## Funding

This article is supported by the following resources: NIH K99 CA237851; T32 CA200561; DOH01-ROWLEY-2019-00037; S10 OD019961 for the use of the Perkin Elmer Scanner; Gruss-Lipper Biophotonics Center and its Integrated Imaging Program at the Albert Einstein College of Medicine (AECOM); Jane A. and Myles P. Dempsey.

## Conflict of Interest

The authors declare that the research was conducted in the absence of any commercial or financial relationships that could be construed as a potential conflict of interest.

## References

[1] Farkona S Diamandis EP Blasutig IM. Cancer immunotherapy: the beginning of the end of cancer? BMC Med (2016) 14:73. 10.1186/s12916-016-0623-5 27151159 PMC4858828

[2] Huang Y Kim BYS Chan CK Hahn SM Weissman IL Jiang W. Improving immune-vascular crosstalk for cancer immunotherapy. Nat Rev Immunol (2018) 18:195–203. 10.1038/nri.2017.145 29332937 PMC5922422

[3] Borst J Ahrends T Babala N Melief CJM Kastenmuller W. CD4(+) T cell help in cancer immunology and immunotherapy. Nat Rev Immunol (2018) 18:635–47. 10.1038/s41577-018-0044-0 30057419

[4] O’Donnell JS Teng MWL Smyth MJ. Cancer immunoediting and resistance to T cell-based immunotherapy. Nat Rev Clin Oncol (2019) 16:151–67. 10.1038/s41571-018-0142-8 30523282

[5] Shimasaki N Jain A Campana D. NK cells for cancer immunotherapy. Nat Rev Drug Discov (2020) 19:200–18. 10.1038/s41573-019-0052-1 31907401

[6] Wculek SK Cueto FJ Mujal AM Melero I Krummel MF Sancho D. Dendritic cells in cancer immunology and immunotherapy. Nat Rev Immunol (2020) 20:7–24. 10.1038/s41577-019-0210-z 31467405

[7] Khalil DN Smith EL Brentjens RJ Wolchok JD. The future of cancer treatment: immunomodulation, CARs and combination immunotherapy. Nat Rev Clin Oncol (2016) 13:273–90. 10.1038/nrclinonc.2016.25 PMC5551685 26977780

[8] Masopust D Schenkel JM. The integration of T cell migration, differentiation and function. Nat Rev Immunol (2013) 13:309–20. 10.1038/nri3442 23598650

[9] Slaney CY Kershaw MH Darcy PK. Trafficking of T cells into tumors. Cancer Res (2014) 74:7168–74. 10.1158/0008-5472.CAN-14-2458 25477332

[10] Speiser DE Ho PC Verdeil G. Regulatory circuits of T cell function in cancer. Nat Rev Immunol (2016) 16:599–611. 10.1038/nri.2016.80 27526640

[11] Adams S Goldstein LJ Sparano JA Demaria S Badve SS. Tumor infiltrating lymphocytes (TILs) improve prognosis in patients with triple negative breast cancer (TNBC). Oncoimmunology (2015) 4:e985930. 10.4161/2162402X.2014.985930 26405612 PMC4570112

[12] Galon J Costes A Sanchez-Cabo F Kirilovsky A Mlecnik B Lagorce-Pages C. Type, density, and location of immune cells within human colorectal tumors predict clinical outcome. Science (2006) 313:1960–4. 10.1126/science.1129139 17008531

[13] Kim ST Jeong H Woo OH Seo JH Kim A Lee ES. Tumor-infiltrating lymphocytes, tumor characteristics, and recurrence in patients with early breast cancer. Am J Clin Oncol (2013) 36:224–31. 10.1097/COC.0b013e3182467d90 22495453

[14] Kmiecik J Poli A Brons NH Waha A Eide GE Enger PO. Elevated CD3+ and CD8+ tumor-infiltrating immune cells correlate with prolonged survival in glioblastoma patients despite integrated immunosuppressive mechanisms in the tumor microenvironment and at the systemic level. J Neuroimmunol (2013) 264:71–83. 10.1016/j.jneuroim.2013.08.013 24045166

[15] Piersma SJ Jordanova ES van Poelgeest MI Kwappenberg KM van der Hulst JM Drijfhout JW. High number of intraepithelial CD8+ tumor-infiltrating lymphocytes is associated with the absence of lymph node metastases in patients with large early-stage cervical cancer. Cancer Res (2007) 67:354–61. 10.1158/0008-5472.CAN-06-3388 17210718

[16] Kim A Lee SJ Ahn J Park WY Shin DH Lee CH. The prognostic significance of tumor-infiltrating lymphocytes assessment with hematoxylin and eosin sections in resected primary lung adenocarcinoma. PLoS One (2019) 14:e0224430. 10.1371/journal.pone.0224430 31743333 PMC6863614

[17] Eerola AK Soini Y Paakko P. A high number of tumor-infiltrating lymphocytes are associated with a small tumor size, low tumor stage, and a favorable prognosis in operated small cell lung carcinoma. Clin Cancer Res (2000) 6:1875–81. 10815910

[18] Tille JC Vieira AF Saint-Martin C Djerroudi L Furhmann L Bidard FC. Tumor-infiltrating lymphocytes are associated with poor prognosis in invasive lobular breast carcinoma. Mod Pathol (2020) 33(11):2198–207. 10.1038/s41379-020-0561-9 32404955

[19] Yildirim N Akman L Acar K Demir S Ozkan S Alan N. Do tumor-infiltrating lymphocytes really indicate favorable prognosis in epithelial ovarian cancer? Eur J Obstet Gynecol Reprod Biol (2017) 215:55–61. 10.1016/j.ejogrb.2017.06.005 28601728

[20] Konig L Mairinger FD Hoffmann O Bittner AK Schmid KW Kimmig R. Dissimilar patterns of tumor-infiltrating immune cells at the invasive tumor front and tumor center are associated with response to neoadjuvant chemotherapy in primary breast cancer. BMC Cancer (2019) 19:120. 10.1186/s12885-019-5320-2 30717704 PMC6360695

[21] Egelston CA Avalos C Tu TY Rosario A Wang R Solomon S. Resident memory CD8+ T cells within cancer islands mediate survival in breast cancer patients. JCI Insight (2019) 4(19):e130000. 10.1172/jci.insight.130000 PMC6795408 31465302

[22] Hida AI Watanabe T Sagara Y Kashiwaba M Sagara Y Aogi K. Diffuse distribution of tumor-infiltrating lymphocytes is a marker for better prognosis and chemotherapeutic effect in triple-negative breast cancer. Breast Cancer Res Treat (2019) 178:283–94. 10.1007/s10549-019-05390-x 31402409

[23] Tao Y Gross N Liu Y Zhang L Li G Huang Z. A high ratio of IL-12Rbeta2-positive tumor-infiltrating lymphocytes indicates favorable prognosis in laryngeal cancer. Oral Oncol (2017) 74:148–56. 10.1016/j.oraloncology.2017.10.006 29103744

[24] Wang B Wu S Zeng H Liu Z Dong W He W. CD103+ Tumor Infiltrating Lymphocytes Predict a Favorable Prognosis in Urothelial Cell Carcinoma of the Bladder. J Urol (2015) 194:556–62. 10.1016/j.juro.2015.02.2941 25752441

[25] Hojo S Koizumi K Tsuneyama K Arita Y Cui Z Shinohara K. High-level expression of chemokine CXCL16 by tumor cells correlates with a good prognosis and increased tumor-infiltrating lymphocytes in colorectal cancer. Cancer Res (2007) 67:4725–31. 10.1158/0008-5472.CAN-06-3424 17510400

[26] Kinoshita F Takada K Yamada Y Oku Y Kosai K Ono Y. Combined Evaluation of Tumor-Infiltrating CD8 + and FoxP3 + Lymphocytes Provides Accurate Prognosis in Stage IA Lung Adenocarcinoma. Ann Surg Oncol (2020) 27:2102–9. 10.1245/s10434-019-08029-9 31773516

[27] Schirosi L Saponaro C Giotta F Popescu O Pastena MI Scarpi E. Tumor Infiltrating Lymphocytes and NHERF1 Impact on Prognosis of Breast Cancer Patients. Transl Oncol (2020) 13:186–92. 10.1016/j.tranon.2019.10.020 PMC6931214 31865181

[28] Sanchez-Canteli M Granda-Diaz R Del Rio-Ibisate N Allonca E Lopez-Alvarez F Agorreta J. PD-L1 expression correlates with tumor-infiltrating lymphocytes and better prognosis in patients with HPV-negative head and neck squamous cell carcinomas. Cancer Immunol Immunother (2020) 69(10):2089–100. 10.1007/s00262-020-02604-w PMC11027666 32448984

[29] Sideras K Biermann K Verheij J Takkenberg BR Mancham S Hansen BE. PD-L1, Galectin-9 and CD8(+) tumor-infiltrating lymphocytes are associated with survival in hepatocellular carcinoma. Oncoimmunology (2017) 6:e1273309. 10.1080/2162402X.2016.1273309 28344887 PMC5353918

[30] Boxberg M Steiger K Lenze U Rechl H von Eisenhart-Rothe R Wortler K. PD-L1 and PD-1 and characterization of tumor-infiltrating lymphocytes in high grade sarcomas of soft tissue - prognostic implications and rationale for immunotherapy. Oncoimmunology (2018) 7:e1389366. 10.1080/2162402X.2017.1389366 29399389 PMC5790346

[31] Dobosz P Dzieciatkowski T. The Intriguing History of Cancer Immunotherapy. Front Immunol (2019) 10:2965. 10.3389/fimmu.2019.02965 31921205 PMC6928196

[32] Burnet FM. Immunological aspects of malignant disease. Lancet (1967) 1:1171–4. 10.1016/S0140-6736(67)92837-1 4165129

[33] Burnet FM. The concept of immunological surveillance. Prog Exp Tumor Res (1970) 13:1–27. 10.1159/000386035 4921480

[34] Maloney DG Grillo-Lopez AJ White CA Bodkin D Schilder RJ Neidhart JA. IDEC-C2B8 (Rituximab) anti-CD20 monoclonal antibody therapy in patients with relapsed low-grade non-Hodgkin’s lymphoma. Blood (1997) 90:2188–95. 10.1182/blood.V90.6.2188 9310469

[35] Reff ME Carner K Chambers KS Chinn PC Leonard JE Raab R. Depletion of B cells in vivo by a chimeric mouse human monoclonal antibody to CD20. Blood (1994) 83:435–45. 10.1182/blood.V83.2.435.435 7506951

[36] Eshhar Z Waks T Gross G Schindler DG. Specific activation and targeting of cytotoxic lymphocytes through chimeric single chains consisting of antibody-binding domains and the gamma or zeta subunits of the immunoglobulin and T-cell receptors. Proc Natl Acad Sci USA (1993) 90:720–4. 10.1073/pnas.90.2.720 PMC45737 8421711

[37] Kochenderfer JN Rosenberg SA. Treating B-cell cancer with T cells expressing anti-CD19 chimeric antigen receptors. Nat Rev Clin Oncol (2013) 10:267–76. 10.1038/nrclinonc.2013.46 PMC6322669 23546520

[38] Kochenderfer JN Wilson WH Janik JE Dudley ME Stetler-Stevenson M Feldman SA. Eradication of B-lineage cells and regression of lymphoma in a patient treated with autologous T cells genetically engineered to recognize CD19. Blood (2010) 116:4099–102. 10.1182/blood-2010-04-281931 PMC2993617 20668228

[39] Morales A Eidinger D Bruce AW. Intracavitary Bacillus Calmette-Guerin in the Treatment of Superficial Bladder Tumors. J Urol (2017) 197:S142–S5. 10.1016/j.juro.2016.10.101 28012770

[40] Olszanski AJ. Principles of immunotherapy. J Natl Compr Canc Netw (2015) 13:670–2. 10.6004/jnccn.2015.0199 25995426

[41] Kalos M. Chimeric antigen receptor-engineered T cells in CLL: the next chapter unfolds. J Immunother Cancer (2016) 4:5. 10.1186/s40425-016-0108-2 26885367 PMC4754893

[42] Kochenderfer JN Dudley ME Kassim SH Somerville RP Carpenter RO Stetler-Stevenson M. Chemotherapy-refractory diffuse large B-cell lymphoma and indolent B-cell malignancies can be effectively treated with autologous T cells expressing an anti-CD19 chimeric antigen receptor. J Clin Oncol (2015) 33:540–9. 10.1200/JCO.2014.56.2025 PMC4322257 25154820

[43] Abbott M Ustoyev Y. Cancer and the Immune System: The History and Background of Immunotherapy. Semin Oncol Nurs (2019) 35:150923. 10.1016/j.soncn.2019.08.002 31526550

[44] Pitt JM Marabelle A Eggermont A Soria JC Kroemer G Zitvogel L. Targeting the tumor microenvironment: removing obstruction to anticancer immune responses and immunotherapy. Ann Oncol (2016) 27:1482–92. 10.1093/annonc/mdw168 27069014

[45] Schietinger A Philip M Krisnawan VE Chiu EY Delrow JJ Basom RS. Tumor-Specific T Cell Dysfunction Is a Dynamic Antigen-Driven Differentiation Program Initiated Early during Tumorigenesis. Immunity (2016) 45:389–401. 10.1016/j.immuni.2016.07.011 27521269 PMC5119632

[46] Schreiber RD Old LJ Smyth MJ. Cancer immunoediting: integrating immunity’s roles in cancer suppression and promotion. Science (2011) 331:1565–70. 10.1126/science.1203486 21436444

[47] Sanchez LR Borriello L Entenberg D Condeelis JS Oktay MH Karagiannis GS. The emerging roles of macrophages in cancer metastasis and response to chemotherapy. J Leukoc Biol (2019) 106:259–74. 10.1002/JLB.MR0218-056RR PMC6779158 30720887

[48] Condamine T Ramachandran I Youn JI Gabrilovich DI. Regulation of tumor metastasis by myeloid-derived suppressor cells. Annu Rev Med (2015) 66:97–110. 10.1146/annurev-med-051013-052304 25341012 PMC4324727

[49] Safarzadeh E Orangi M Mohammadi H Babaie F Baradaran B. Myeloid-derived suppressor cells: Important contributors to tumor progression and metastasis. J Cell Physiol (2018) 233:3024–36. 10.1002/jcp.26075 28661031

[50] El-Kenawi A Hanggi K Ruffell B. The Immune Microenvironment and Cancer Metastasis. Cold Spring Harb Perspect Med (2020) 10:a037424. 10.1101/cshperspect.a037424 PMC7117953 31501262

[51] Kadioglu E De Palma M. Cancer Metastasis: Perivascular Macrophages Under Watch. Cancer Discov (2015) 5:906–8. 10.1158/2159-8290.CD-15-0819 26334045

[52] Rakic A Beaudry P Mahoney DJ. The complex interplay between neutrophils and cancer. Cell Tissue Res (2018) 371:517–29. 10.1007/s00441-017-2777-7 29427051

[53] Chouaib S Noman MZ Kosmatopoulos K Curran MA. Hypoxic stress: obstacles and opportunities for innovative immunotherapy of cancer. Oncogene (2017) 36:439–45. 10.1038/onc.2016.225 PMC5937267 27345407

[54] Padera TP Stoll BR Tooredman JB Capen D di Tomaso E Jain RK. Pathology: cancer cells compress intratumour vessels. Nature (2004) 427:695. 10.1038/427695a 14973470

[55] Welti J Loges S Dimmeler S Carmeliet P. Recent molecular discoveries in angiogenesis and antiangiogenic therapies in cancer. J Clin Invest (2013) 123:3190–200. 10.1172/JCI70212 PMC3726176 23908119

[56] Schaaf MB Garg AD Agostinis P. Defining the role of the tumor vasculature in antitumor immunity and immunotherapy. Cell Death Dis (2018) 9:115. 10.1038/s41419-017-0061-0 29371595 PMC5833710

[57] Steinbrink K Graulich E Kubsch S Knop J Enk AH. CD4(+) and CD8(+) anergic T cells induced by interleukin-10-treated human dendritic cells display antigen-specific suppressor activity. Blood (2002) 99:2468–76. 10.1182/blood.V99.7.2468 11895781

[58] Tormoen GW Crittenden MR Gough MJ. Role of the immunosuppressive microenvironment in immunotherapy. Adv Radiat Oncol (2018) 3:520–6. 10.1016/j.adro.2018.08.018 PMC6200899 30370351

[59] Vonderheide RH. The Immune Revolution: A Case for Priming, Not Checkpoint. Cancer Cell (2018) 33:563–9. 10.1016/j.ccell.2018.03.008 PMC5898647 29634944

[60] Xu J Escamilla J Mok S David J Priceman S West B. CSF1R signaling blockade stanches tumor-infiltrating myeloid cells and improves the efficacy of radiotherapy in prostate cancer. Cancer Res (2013) 73:2782–94. 10.1158/0008-5472.CAN-12-3981 PMC4097014 23418320

[61] Nagaraj S Gupta K Pisarev V Kinarsky L Sherman S Kang L. Altered recognition of antigen is a mechanism of CD8+ T cell tolerance in cancer. Nat Med (2007) 13:828–35. 10.1038/nm1609 PMC2135607 17603493

[62] Noman MZ Desantis G Janji B Hasmim M Karray S Dessen P. PD-L1 is a novel direct target of HIF-1alpha, and its blockade under hypoxia enhanced MDSC-mediated T cell activation. J Exp Med (2014) 211:781–90. 10.1084/jem.20131916 PMC4010891 24778419

[63] Kusmartsev S Eruslanov E Kubler H Tseng T Sakai Y Su Z. Oxidative stress regulates expression of VEGFR1 in myeloid cells: link to tumor-induced immune suppression in renal cell carcinoma. J Immunol (2008) 181:346–53. 10.4049/jimmunol.181.1.346 18566400

[64] Martinez FO Sica A Mantovani A Locati M. Macrophage activation and polarization. Front Biosci (2008) 13:453–61. 10.2741/2692 17981560

[65] Buckanovich RJ Facciabene A Kim S Benencia F Sasaroli D Balint K. Endothelin B receptor mediates the endothelial barrier to T cell homing to tumors and disables immune therapy. Nat Med (2008) 14:28–36. 10.1038/nm1699 18157142

[66] Motz GT Santoro SP Wang LP Garrabrant T Lastra RR Hagemann IS. Tumor endothelium FasL establishes a selective immune barrier promoting tolerance in tumors. Nat Med (2014) 20:607–15. 10.1038/nm.3541 PMC4060245 24793239

[67] Zang X Sullivan PS Soslow RA Waitz R Reuter VE Wilton A. Tumor associated endothelial expression of B7-H3 predicts survival in ovarian carcinomas. Mod Pathol (2010) 23:1104–12. 10.1038/modpathol.2010.95 PMC2976590 20495537

[68] Zhang L Conejo-Garcia JR Katsaros D Gimotty PA Massobrio M Regnani G. Intratumoral T cells, recurrence, and survival in epithelial ovarian cancer. N Engl J Med (2003) 348:203–13. 10.1056/NEJMoa020177 12529460

[69] Sharma P Allison JP. The future of immune checkpoint therapy. Science (2015) 348:56–61. 10.1126/science.aaa8172 25838373

[70] Morgan RA. Risky business: target choice in adoptive cell therapy. Blood (2013) 122:3392–4. 10.1182/blood-2013-09-527622 PMC3829111 24235126

[71] Turtle CJ Hanafi LA Berger C Hudecek M Pender B Robinson E. Immunotherapy of non-Hodgkin’s lymphoma with a defined ratio of CD8+ and CD4+ CD19-specific chimeric antigen receptor-modified T cells. Sci Transl Med (2016) 8:355ra116. 10.1126/scitranslmed.aaf8621 PMC5045301 27605551

[72] Chapuis AG Ragnarsson GB Nguyen HN Chaney CN Pufnock JS Schmitt TM. Transferred WT1-reactive CD8+ T cells can mediate antileukemic activity and persist in post-transplant patients. Sci Transl Med (2013) 5:174ra27. 10.1126/scitranslmed.3004916 PMC3678970 23447018

[73] Karagiannis GS Condeelis JS Oktay MH. Chemotherapy-Induced Metastasis: Molecular Mechanisms, Clinical Manifestations, Therapeutic Interventions. Cancer Res (2019) 79:4567–76. 10.1158/0008-5472.CAN-19-1147 PMC6744993 31431464

[74] Karagiannis GS Condeelis JS Oktay MH. Chemotherapy-induced metastasis: mechanisms and translational opportunities. Clin Exp Metastasis (2018) 35:269–84. 10.1007/s10585-017-9870-x PMC6035114 29307118

[75] Karagiannis GS Goswami S Jones JG Oktay MH Condeelis JS. Signatures of breast cancer metastasis at a glance. J Cell Sci (2016) 129:1751–8. 10.1242/jcs.183129 PMC4893654 27084578

[76] Hanahan D Weinberg RA. Hallmarks of cancer: the next generation. Cell (2011) 144:646–74. 10.1016/j.cell.2011.02.013 21376230

[77] Zhang SY Song XY Li Y Ye LL Zhou Q Yang WB. Tumor-associated macrophages: A promising target for a cancer immunotherapeutic strategy. Pharmacol Res (2020) 161:105111. 10.1016/j.phrs.2020.105111 33065284

[78] Chaffer CL Weinberg RA. A perspective on cancer cell metastasis. Science (2011) 331:1559–64. 10.1126/science.1203543 21436443

[79] Shibue T Weinberg RA. Metastatic colonization: settlement, adaptation and propagation of tumor cells in a foreign tissue environment. Semin Cancer Biol (2011) 21:99–106. 10.1016/j.semcancer.2010.12.003 21145969

[80] Valastyan S Weinberg RA. Tumor metastasis: molecular insights and evolving paradigms. Cell (2011) 147:275–92. 10.1016/j.cell.2011.09.024 PMC3261217 22000009

[81] Dasgupta A Lim AR Ghajar CM. Circulating and disseminated tumor cells: harbingers or initiators of metastasis? Mol Oncol (2017) 11:40–61. 10.1002/1878-0261.12022 28085223 PMC5423226

[82] Lambert AW Pattabiraman DR Weinberg RA. Emerging Biological Principles of Metastasis. Cell (2017) 168:670–91. 10.1016/j.cell.2016.11.037 PMC5308465 28187288

[83] Turajlic S Swanton C. Metastasis as an evolutionary process. Science (2016) 352:169–75. 10.1126/science.aaf2784 27124450

[84] Seyfried TN Huysentruyt LC. On the origin of cancer metastasis. Crit Rev Oncog (2013) 18:43–73. 10.1615/CritRevOncog.v18.i1-2.40 23237552 PMC3597235

[85] Chaffer CL San Juan BP Lim E Weinberg RA. EMT, cell plasticity and metastasis. Cancer metastasis Rev (2016) 35:645–54. 10.1007/s10555-016-9648-7 27878502

[86] Quail DF Joyce JA. Microenvironmental regulation of tumor progression and metastasis. Nat Med (2013) 19:1423–37. 10.1038/nm.3394 PMC3954707 24202395

[87] Bragado P Sosa MS Keely P Condeelis J Aguirre-Ghiso JA. Microenvironments dictating tumor cell dormancy. Recent results in cancer research Fortschritte der Krebsforschung Progres dans les recherches sur le cancer. Recent Results Cancer Res (2012) 195:25–39. 10.1007/978-3-642-28160-0_3 22527492 PMC3516301

[88] Hanahan D Coussens LM. Accessories to the crime: functions of cells recruited to the tumor microenvironment. Cancer Cell (2012) 21:309–22. 10.1016/j.ccr.2012.02.022 22439926

[89] Roussos ET Condeelis JS Patsialou A. Chemotaxis in cancer. Nat Rev Cancer (2011) 11:573–87. 10.1038/nrc3078 PMC4030706 21779009

[90] Joyce JA Pollard JW. Microenvironmental regulation of metastasis. Nat Rev Cancer (2009) 9:239–52. 10.1038/nrc2618 PMC3251309 19279573

[91] Kaplan RN Rafii S Lyden D. Preparing the “soil”: the premetastatic niche. Cancer Res (2006) 66:11089–93. 10.1158/0008-5472.CAN-06-2407 PMC2952469 17145848

[92] Yamaguchi H Wyckoff J Condeelis J. Cell migration in tumors. Curr Opin Cell Biol (2005) 17:559–64. 10.1016/j.ceb.2005.08.002 16098726

[93] Oktay MH Jones JG. TMEM: a novel breast cancer dissemination marker for the assessment of metastatic risk. Biomark Med (2015) 9:81–4. 10.2217/bmm.14.104 25689896

[94] Robinson BD Sica GL Liu YF Rohan TE Gertler FB Condeelis JS. Tumor microenvironment of metastasis in human breast carcinoma: a potential prognostic marker linked to hematogenous dissemination. Clin Cancer Res (2009) 15:2433–41. 10.1158/1078-0432.CCR-08-2179 PMC3156570 19318480

[95] Rohan TE Xue X Lin HM D’Alfonso TM Ginter PS Oktay MH. Tumor microenvironment of metastasis and risk of distant metastasis of breast cancer. J Natl Cancer Institute (2014) 106(8):dju136. 10.1093/jnci/dju136 PMC4133559 24895374

[96] Sparano JA Gray R Oktay MH Entenberg D Rohan T Xue X. A metastasis biomarker (MetaSite Breast Score) is associated with distant recurrence in hormone receptor-positive, HER2-negative early-stage breast cancer. Nat PJ Breast Cancer (2017) 3:42. 10.1038/s41523-017-0043-5 PMC5678158 29138761

[97] Entenberg D Voiculescu S Guo P Borriello L Wang Y Karagiannis GS. A permanent window for the murine lung enables high-resolution imaging ofcancer metastasis. Nat Methods (2018) 15:73–80. 10.1038/nmeth.4511 29176592 PMC5755704

[98] Borriello L Karagiannis GS Duran CL Coste A Oktay MH Entenberg D. The role of the tumor microenvironment in tumor cell intravasation and dissemination. Eur J Cell Biol (2020) 99:151098. 10.1016/j.ejcb.2020.151098 32800278 PMC7431678

[99] Coste A Karagiannis GS Wang Y Xue EA Lin Y Skobe M. Hematogenous Dissemination of Breast Cancer Cells From Lymph Nodes Is Mediated by Tumor MicroEnvironment of Metastasis Doorways. Front Oncol (2020) 10:571100. 10.3389/fonc.2020.571100 33194666 PMC7649363

[100] Goswami S Philippar U Sun D Patsialou A Avraham J Wang W. Identification of invasion specific splice variants of the cytoskeletal protein Mena present in mammary tumor cells during invasion in vivo. Clin Exp Metastasis (2009) 26:153–9. 10.1007/s10585-008-9225-8 PMC3042857 18985426

[101] Balsamo M Mondal C Carmona G McClain LM Riquelme DN Tadros J. The alternatively-included 11a sequence modifies the effects of Mena on actin cytoskeletal organization and cell behavior. Sci Rep (2016) 6:35298. 10.1038/srep35298 27748415 PMC5066228

[102] Tanaka N Yoshida H Suzuki Y Harigaya K. Relative expression of hMena11a and hMenaINV splice isoforms is a useful biomarker in development and progression of human breast carcinoma. Int J Oncol (2014) 45:1921–8. 10.3892/ijo.2014.2591 25109497

[103] Roussos ET Goswami S Balsamo M Wang Y Stobezki R Adler E. Mena invasive (Mena(INV)) and Mena11a isoforms play distinct roles in breast cancer cell cohesion and association with TMEM. Clin Exp Metastasis (2011) 28:515–27. 10.1007/s10585-011-9388-6 PMC3459587 21484349

[104] Roussos ET Balsamo M Alford SK Wyckoff JB Gligorijevic B Wang Y. Mena invasive (MenaINV) promotes multicellular streaming motility and transendothelial migration in a mouse model of breast cancer. J Cell Sci (2011) 124:2120–31. 10.1242/jcs.086231 PMC3113666 21670198

[105] Bear JE Gertler FB. Ena/VASP: towards resolving a pointed controversy at the barbed end. J Cell Sci (2009) 122:1947–53. 10.1242/jcs.038125 PMC2723151 19494122

[106] Gertler F Condeelis J. Metastasis: tumor cells becoming MENAcing. Trends Cell Biol (2011) 21:81–90. 10.1016/j.tcb.2010.10.001 21071226 PMC3402095

[107] Philippar U Roussos ET Oser M Yamaguchi H Kim HD Giampieri S. A Mena invasion isoform potentiates EGF-induced carcinoma cell invasion and metastasis. Dev Cell (2008) 15:813–28. 10.1016/j.devcel.2008.09.003 PMC2637261 19081071

[108] Eddy RJ Weidmann MD Sharma VP Condeelis JS. Tumor Cell Invadopodia: Invasive Protrusions that Orchestrate Metastasis. Trends Cell Biol (2017) 27:595–607. 10.1016/j.tcb.2017.03.003 28412099 PMC5524604

[109] Oudin MJ Hughes SK Rohani N Moufarrej MN Jones JG Condeelis JS. Characterization of the expression of the pro-metastatic Mena(INV) isoform during breast tumor progression. Clin Exp Metastasis (2016) 33:249–61. 10.1007/s10585-015-9775-5 PMC4777680 26680363

[110] Weidmann MD Surve CR Eddy RJ Chen X Gertler FB Sharma VP. MenaINV dysregulates cortactin phosphorylation to promote invadopodium maturation. Sci Rep (2016) 6:36142. 10.1038/srep36142 27824079 PMC5099927

[111] Rajadurai CV Havrylov S Coelho PP Ratcliffe CD Zaoui K Huang BH. 5’-Inositol phosphatase SHIP2 recruits Mena to stabilize invadopodia for cancer cell invasion. J Cell Biol (2016) 214:719–34. 10.1083/jcb.201501003 PMC5021089 27597754

[112] Oudin MJ Miller MA Klazen JA Kosciuk T Lussiez A Hughes SK. MenaINV mediates synergistic cross-talk between signaling pathways driving chemotaxis and haptotaxis. Mol Biol Cell (2016) 27:3085–94. 10.1091/mbc.e16-04-0212 PMC5063616 27559126

[113] Lin L Yang XM Li J Zhang YL Qin W Zhang ZG. Microfilament regulatory protein MENA increases activity of RhoA and promotes metastasis of hepatocellular carcinoma. Exp Cell Res (2014) 327:113–22. 10.1016/j.yexcr.2014.05.010 24859350

[114] Pignatelli J Goswami S Jones JG Rohan TE Pieri E Chen X. Invasive breast carcinoma cells from patients exhibit MenaINV- and macrophage-dependent transendothelial migration. Sci Signaling (2014) 7:ra112. 10.1126/scisignal.2005329 PMC4266931 25429076

[115] Aiello NM Kang Y. Context-dependent EMT programs in cancer metastasis. J Exp Med (2019) 216(5):1016–26. 10.1084/jem.20181827 PMC6504222 30975895

[116] Bronsert P Enderle-Ammour K Bader M Timme S Kuehs M Csanadi A. Cancer cell invasion and EMT marker expression: a three-dimensional study of the human cancer-host interface. J Pathol (2014) 234:410–22. 10.1002/path.4416 25081610

[117] Kalluri R. EMT: when epithelial cells decide to become mesenchymal-like cells. J Clin Invest (2009) 119:1417–9. 10.1172/JCI39675 PMC2689122 19487817

[118] Kalluri R Weinberg RA. The basics of epithelial-mesenchymal transition. J Clin Invest (2009) 119:1420–8. 10.1172/JCI39104 PMC2689101 19487818

[119] Patsialou A Bravo-Cordero JJ Wang Y Entenberg D Liu H Clarke M. Intravital multiphoton imaging reveals multicellular streaming as a crucial component of in vivo cell migration in human breast tumors. Intravital (2013) 2:e25294. 10.4161/intv.25294 25013744 PMC3908591

[120] Sharma VP Beaty BT Patsialou A Liu H Clarke M Cox D. Reconstitution of in vivo macrophage-tumor cell pairing and streaming motility on one-dimensional micro-patterned substrates. Intravital (2012) 1:77–85. 10.4161/intv.22054 24634804 PMC3908597

[121] Goswami S Sahai E Wyckoff JB Cammer M Cox D Pixley FJ. Macrophages promote the invasion of breast carcinoma cells via a colony-stimulating factor-1/epidermal growth factor paracrine loop. Cancer Res (2005) 65:5278–83. 10.1158/0008-5472.CAN-04-1853 15958574

[122] Hernandez L Smirnova T Kedrin D Wyckoff J Zhu L Stanley ER. The EGF/CSF-1 paracrine invasion loop can be triggered by heregulin beta1 and CXCL12. Cancer Res (2009) 69:3221–7. 10.1158/0008-5472.CAN-08-2871 PMC2820720 19293185

[123] Patsialou A Wyckoff J Wang Y Goswami S Stanley ER Condeelis JS. Invasion of human breast cancer cells in vivo requires both paracrine and autocrine loops involving the colony-stimulating factor-1 receptor. Cancer Res (2009) 69:9498–506. 10.1158/0008-5472.CAN-09-1868 PMC2794986 19934330

[124] Pignatelli J Bravo-Cordero JJ Roh-Johnson M Gandhi SJ Wang Y Chen X. Macrophage-dependent tumor cell transendothelial migration is mediated by Notch1/MenaINV-initiated invadopodium formation. Sci Rep (2016) 6:37874. 10.1038/srep37874 27901093 PMC5129016

[125] Roh-Johnson M Bravo-Cordero JJ Patsialou A Sharma VP Guo P Liu H. Macrophage contact induces RhoA GTPase signaling to trigger tumor cell intravasation. Oncogene (2014) 33:4203–12. 10.1038/onc.2013.377 PMC3962803 24056963

[126] Leung E Xue A Wang Y Rougerie P Sharma VP Eddy R. Blood vessel endothelium-directed tumor cell streaming in breast tumors requires the HGF/C-Met signaling pathway. Oncogene (2017) 36:2680–92. 10.1038/onc.2016.421 PMC5426963 27893712

[127] Jin F Brockmeier U Otterbach F Metzen E. New insight into the SDF-1/CXCR4 axis in a breast carcinoma model: hypoxia-induced endothelial SDF-1 and tumor cell CXCR4 are required for tumor cell intravasation. Mol Cancer Res MCR (2012) 10:1021–31. 10.1158/1541-7786.MCR-11-0498 22767589

[128] Harney AS Arwert EN Entenberg D Wang Y Guo P Qian BZ. Real-Time Imaging Reveals Local, Transient Vascular Permeability, and Tumor Cell Intravasation Stimulated by TIE2hi Macrophage-Derived VEGFA. Cancer Discov (2015) 5:932–43. 10.1158/2159-8290.CD-15-0012 PMC4560669 26269515

[129] Fang Z Wen C Chen X Yin R Zhang C Wang X. Myeloid-derived suppressor cell and macrophage exert distinct angiogenic and immunosuppressive effects in breast cancer. Oncotarget (2017) 8:54173–86. 10.18632/oncotarget.17013 PMC5589571 28903332

[130] Kumar V Patel S Tcyganov E Gabrilovich DI. The Nature of Myeloid-Derived Suppressor Cells in the Tumor Microenvironment. Trends Immunol (2016) 37:208–20. 10.1016/j.it.2016.01.004 PMC4775398 26858199

[131] Mitchem JB Brennan DJ Knolhoff BL Belt BA Zhu Y Sanford DE. Targeting tumor-infiltrating macrophages decreases tumor-initiating cells, relieves immunosuppression, and improves chemotherapeutic responses. Cancer Res (2013) 73:1128–41. 10.1158/0008-5472.CAN-12-2731 PMC3563931 23221383

[132] Gajewski TF Meng Y Harlin H. Immune suppression in the tumor microenvironment. J Immunother (2006) 29:233–40. 10.1097/01.cji.0000199193.29048.56 16699366

[133] Zhao Y Rahmy S Liu Z Zhang C Lu X. Rational targeting of immunosuppressive neutrophils in cancer. Pharmacol Ther (2020) 212:107556. 10.1016/j.pharmthera.2020.107556 32343986

[134] Trovato R Cane S Petrova V Sartoris S Ugel S De Sanctis F. The Engagement Between MDSCs and Metastases: Partners in Crime. Front Oncol (2020) 10:165. 10.3389/fonc.2020.00165 32133298 PMC7040035

[135] Lebegge E Arnouk SM Bardet PMR Kiss M Raes G Van Ginderachter JA. Innate Immune Defense Mechanisms by Myeloid Cells That Hamper Cancer Immunotherapy. Front Immunol (2020) 11:1395. 10.3389/fimmu.2020.01395 32733461 PMC7363805

[136] Furumaya C Martinez-Sanz P Bouti P Kuijpers TW Matlung HL. Plasticity in Pro- and Anti-tumor Activity of Neutrophils: Shifting the Balance. Front Immunol (2020) 11:2100. 10.3389/fimmu.2020.02100 32983165 PMC7492657

[137] Wang Y Jia A Bi Y Wang Y Yang Q Cao Y. Targeting Myeloid-Derived Suppressor Cells in Cancer Immunotherapy. Cancers (Basel) (2020) 12(9):2626. 10.3390/cancers12092626 PMC7564060 32942545

[138] Lin EY Jones JG Li P Zhu L Whitney KD Muller WJ. Progression to malignancy in the polyoma middle T oncoprotein mouse breast cancer model provides a reliable model for human diseases. Am J Pathol (2003) 163:2113–26. 10.1016/S0002-9440(10)63568-7 PMC1892434 14578209

[139] Karagiannis GS Pastoriza JM Wang Y Harney AS Entenberg D Pignatelli J. Neoadjuvant chemotherapy induces breast cancer metastasis through a TMEM-mediated mechanism. Sci Trans Med (2017) 9(397):eaan0026. 10.1126/scitranslmed.aan0026 PMC5592784 28679654

[140] Hughes R Qian BZ Rowan C Muthana M Keklikoglou I Olson OC. Perivascular M2 Macrophages Stimulate Tumor Relapse after Chemotherapy. Cancer Res (2015) 75:3479–91. 10.1158/0008-5472.CAN-14-3587 PMC5024531 26269531

[141] Volk-Draper L Hall K Griggs C Rajput S Kohio P DeNardo D. Paclitaxel therapy promotes breast cancer metastasis in a TLR4-dependent manner. Cancer Res (2014) 74:5421–34. 10.1158/0008-5472.CAN-14-0067 PMC4185415 25274031

[142] Chang YS Jalgaonkar SP Middleton JD Hai T. Stress-inducible gene Atf3 in the noncancer host cells contributes to chemotherapy-exacerbated breast cancer metastasis. Proc Natl Acad Sci USA (2017) 114:E7159–E68. 10.1073/pnas.1700455114 PMC5576783 28784776

[143] Shree T Olson OC Elie BT Kester JC Garfall AL Simpson K. Macrophages and cathepsin proteases blunt chemotherapeutic response in breast cancer. Genes Dev (2011) 25:2465–79. 10.1101/gad.180331.111 PMC3243057 22156207

[144] Daenen LG Roodhart JM van Amersfoort M Dehnad M Roessingh W Ulfman LH. Chemotherapy enhances metastasis formation via VEGFR-1-expressing endothelial cells. Cancer Res (2011) 71:6976–85. 10.1158/0008-5472.CAN-11-0627 21975929

[145] Liu KX Joshi S. “Re-educating” Tumor Associated Macrophages as a Novel Immunotherapy Strategy for Neuroblastoma. Front Immunol (2020) 11:1947. 10.3389/fimmu.2020.01947 32983125 PMC7493646

[146] Gauttier V Pengam S Durand J Biteau K Mary C Morello A. Selective SIRPalpha blockade reverses tumor T cell exclusion and overcomes cancer immunotherapy resistance. J Clin Invest (2020) 130:6109–23. 10.1172/JCI135528 PMC7598080 33074246

[147] Lowe KL Cole D Kenefeck R OK I Lepore M Jakobsen BK. Novel TCR-based biologics: mobilising T cells to warm ‘cold’ tumours. Cancer Treat Rev (2019) 77:35–43. 10.1016/j.ctrv.2019.06.001 31207478

[148] Kong X. Discovery of New Immune Checkpoints: Family Grows Up. Adv Exp Med Biol (2020) 1248:61–82. 10.1007/978-981-15-3266-5_4 32185707

[149] Mlynska A Vaisnore R Rafanavicius V Jocys S Janeiko J Petrauskyte M. A gene signature for immune subtyping of desert, excluded, and inflamed ovarian tumors. Am J Reprod Immunol (2020) 84:e13244. 10.1111/aji.13244 32294293

[150] Job S Rapoud D Dos Santos A Gonzalez P Desterke C Pascal G. Identification of four immune subtypes characterized by distinct composition and functions of tumor microenvironment in intrahepatic cholangiocarcinoma. Hepatology (2019) 72(3):965–81. 10.1002/hep.31092 PMC7589418 31875970

[151] Anderson KG Stromnes IM Greenberg PD. Obstacles Posed by the Tumor Microenvironment to T cell Activity: A Case for Synergistic Therapies. Cancer Cell (2017) 31:311–25. 10.1016/j.ccell.2017.02.008 PMC5423788 28292435

[152] Schenkel JM Masopust D. Identification of a resident T-cell memory core transcriptional signature. Immunol Cell Biol (2014) 92:8–9. 10.1038/icb.2013.67 24165982

[153] Tanaka H Tanaka J Kjaergaard J Shu S. Depletion of CD4+ CD25+ regulatory cells augments the generation of specific immune T cells in tumor-draining lymph nodes. J Immunother (2002) 25:207–17. 10.1097/00002371-200205000-00003 12000862

[154] Nagy JA Chang SH Dvorak AM Dvorak HF. Why are tumour blood vessels abnormal and why is it important to know? Br J Cancer (2009) 100:865–9. 10.1038/sj.bjc.6604929 PMC2661770 19240721

[155] Nagy JA Chang SH Shih SC Dvorak AM Dvorak HF. Heterogeneity of the tumor vasculature. Semin Thromb Hemost (2010) 36:321–31. 10.1055/s-0030-1253454 PMC3278036 20490982

[156] Suzuki T Yanagi K Ookawa K Hatakeyama K Ohshima N. Flow visualization of microcirculation in solid tumor tissues: intravital microscopic observation of blood circulation by use of a confocal laser scanning microscope. Front Med Biol Eng (1996) 7:253–63. 8956966

[157] Baluk P Morikawa S Haskell A Mancuso M McDonald DM. Abnormalities of basement membrane on blood vessels and endothelial sprouts in tumors. Am J Pathol (2003) 163:1801–15. 10.1016/S0002-9440(10)63540-7 PMC1892429 14578181

[158] Baluk P Hashizume H McDonald DM. Cellular abnormalities of blood vessels as targets in cancer. Curr Opin Genet Dev (2005) 15:102–11. 10.1016/j.gde.2004.12.005 15661540

[159] Raza A Franklin MJ Dudek AZ. Pericytes and vessel maturation during tumor angiogenesis and metastasis. Am J Hematol (2010) 85:593–8. 10.1002/ajh.21745 20540157

[160] Bergers G Song S Meyer-Morse N Bergsland E Hanahan D. Benefits of targeting both pericytes and endothelial cells in the tumor vasculature with kinase inhibitors. J Clin Invest (2003) 111:1287–95. 10.1172/JCI200317929 PMC154450 12727920

[161] Song S Ewald AJ Stallcup W Werb Z Bergers G. PDGFRbeta+ perivascular progenitor cells in tumours regulate pericyte differentiation and vascular survival. Nat Cell Biol (2005) 7:870–9. 10.1038/ncb1288 PMC2771163 16113679

[162] Bergers G Song S. The role of pericytes in blood-vessel formation and maintenance. Neuro Oncol (2005) 7:452–64. 10.1215/S1152851705000232 PMC1871727 16212810

[163] Ager A. Lymphocyte recirculation and homing: roles of adhesion molecules and chemoattractants. Trends Cell Biol (1994) 4:326–33. 10.1016/0962-8924(94)90234-8 14731470

[164] Steeber DA Tedder TF. Adhesion molecule cascades direct lymphocyte recirculation and leukocyte migration during inflammation. Immunol Res (2000) 22:299–317. 10.1385/IR:22:2-3:299 11339364

[165] Tedder TF Steeber DA Chen A Engel P. The selectins: vascular adhesion molecules. FASEB J (1995) 9:866–73. 10.1096/fasebj.9.10.7542213 7542213

[166] Yong K Khwaja A. Leucocyte cellular adhesion molecules. Blood Rev (1990) 4:211–25. 10.1016/0268-960X(90)90001-9 1706206

[167] Issekutz TB. Lymphocyte homing to sites of inflammation. Curr Opin Immunol (1992) 4:287–93. 10.1016/0952-7915(92)90078-S 1418707

[168] Faveeuw C Preece G Ager A. Transendothelial migration of lymphocytes across high endothelial venules into lymph nodes is affected by metalloproteinases. Blood (2001) 98:688–95. 10.1182/blood.V98.3.688 11468168

[169] Indrasingh I Chandi G Vettivel S. Route of lymphocyte migration through the high endothelial venule (HEV) in human palatine tonsil. Ann Anat (2002) 184:77–84. 10.1016/S0940-9602(02)80040-1 11876486

[170] Azzali G Arcari ML Caldara GF Vitale M. The “intraendothelial canalicular formation”: the route for lymphocyte diapedesis at the level of peripheral and mucosa-associated lymphoid tissue HEVs. Acta BioMed (2010) 81:5–20. 20857848

[171] Bai Z Cai L Umemoto E Takeda A Tohya K Komai Y. Constitutive lymphocyte transmigration across the basal lamina of high endothelial venules is regulated by the autotaxin/lysophosphatidic acid axis. J Immunol (2013) 190:2036–48. 10.4049/jimmunol.1202025 23365076

[172] Bellone M Calcinotto A. Ways to enhance lymphocyte trafficking into tumors and fitness of tumor infiltrating lymphocytes. Front Oncol (2013) 3:231. 10.3389/fonc.2013.00231 24062984 PMC3769630

[173] Tseng JF Willett CG Fernandez-del Castillo C Ryan DP Clark JW Zhu AX. Patients undergoing treatment for pancreatic adenocarcinoma can mount an effective immune response to vaccinations. Pancreatology (2005) 5:67–74. 10.1159/000084492 15775701

[174] Prazeres P Almeida VM Lousado L Andreotti JP Paiva AE Santos GSP. Macrophages Generate Pericytes in the Developing Brain. Cell Mol Neurobiol (2018) 38:777–82. 10.1007/s10571-017-0549-2 PMC6180321 28894964

[175] Stallcup WB. The NG2 Proteoglycan in Pericyte Biology. Adv Exp Med Biol (2018) 1109:5–19. 10.1007/978-3-030-02601-1_2 30523586

[176] Yang Y Andersson P Hosaka K Zhang Y Cao R Iwamoto H. The PDGF-BB-SOX7 axis-modulated IL-33 in pericytes and stromal cells promotes metastasis through tumour-associated macrophages. Nat Commun (2016) 7:11385. 10.1038/ncomms11385 27150562 PMC4859070

[177] Tattersall IW Du J Cong Z Cho BS Klein AM Dieck CL. In vitro modeling of endothelial interaction with macrophages and pericytes demonstrates Notch signaling function in the vascular microenvironment. Angiogenesis (2016) 19:201–15. 10.1007/s10456-016-9501-1 PMC5576170 26965898

[178] Stallcup WB You WK Kucharova K Cejudo-Martin P Yotsumoto F. NG2 Proteoglycan-Dependent Contributions of Pericytes and Macrophages to Brain Tumor Vascularization and Progression. Microcirculation (2016) 23:122–33. 10.1111/micc.12251 PMC4744154 26465118

[179] Zhu C Chrifi I Mustafa D van der Weiden M Leenen PJM Duncker DJ. CECR1-mediated cross talk between macrophages and vascular mural cells promotes neovascularization in malignant glioma. Oncogene (2017) 36:5356–68. 10.1038/onc.2017.145 PMC5611481 28534507

[180] Schmitz V Vilanueva H Raskopf E Hilbert T Barajas M Dzienisowicz C. Increased VEGF levels induced by anti-VEGF treatment are independent of tumor burden in colorectal carcinomas in mice. Gene Ther (2006) 13:1198–205. 10.1038/sj.gt.3302772 16617302

[181] Bergers G Hanahan D. Modes of resistance to anti-angiogenic therapy. Nat Rev Cancer (2008) 8:592–603. 10.1038/nrc2442 18650835 PMC2874834

[182] Saharinen P Eklund L Pulkki K Bono P Alitalo K. VEGF and angiopoietin signaling in tumor angiogenesis and metastasis. Trends Mol Med (2011) 17:347–62. 10.1016/j.molmed.2011.01.015 21481637

[183] Jayson GC Kerbel R Ellis LM Harris AL. Antiangiogenic therapy in oncology: current status and future directions. Lancet (2016) 388:518–29. 10.1016/S0140-6736(15)01088-0 26853587

[184] Zarrin B Zarifi F Vaseghi G Javanmard SH. Acquired tumor resistance to antiangiogenic therapy: Mechanisms at a glance. J Res Med Sci (2017) 22:117. 10.4103/jrms.JRMS_182_17 29184575 PMC5680657

[185] Baeriswyl V Christofori G. The angiogenic switch in carcinogenesis. Semin Cancer Biol (2009) 19:329–37. 10.1016/j.semcancer.2009.05.003 19482086

[186] Bergers G Benjamin LE. Tumorigenesis and the angiogenic switch. Nat Rev Cancer (2003) 3:401–10. 10.1038/nrc1093 12778130

[187] Sitohy B Nagy JA Jaminet SC Dvorak HF. Tumor-surrogate blood vessel subtypes exhibit differential susceptibility to anti-VEGF therapy. Cancer Res (2011) 71:7021–8. 10.1158/0008-5472.CAN-11-1693 PMC3217088 21937680

[188] Lewis CE Harney AS Pollard JW. The Multifaceted Role of Perivascular Macrophages in Tumors. Cancer Cell (2016) 30:18–25. 10.1016/j.ccell.2016.05.017 27411586 PMC5024543

[189] Ferrara N. Role of myeloid cells in vascular endothelial growth factor-independent tumor angiogenesis. Curr Opin Hematol (2010) 17:219–24. 10.1097/MOH.0b013e3283386660 20308892

[190] Riabov V Gudima A Wang N Mickley A Orekhov A Kzhyshkowska J. Role of tumor associated macrophages in tumor angiogenesis and lymphangiogenesis. Front Physiol (2014) 5:75. 10.3389/fphys.2014.00075 24634660 PMC3942647

[191] Bates DO. Vascular endothelial growth factors and vascular permeability. Cardiovasc Res (2010) 87:262–71. 10.1093/cvr/cvq105 PMC2895541 20400620

[192] Bates DO Lodwick D Williams B. Vascular endothelial growth factor and microvascular permeability. Microcirculation (1999) 6:83–96. 10.1111/j.1549-8719.1999.tb00091.x 10466111

[193] Lee YC. The involvement of VEGF in endothelial permeability: a target for anti-inflammatory therapy. Curr Opin Invest Drugs (2005) 6:1124–30. 16312133

[194] Roberts WG Palade GE. Increased microvascular permeability and endothelial fenestration induced by vascular endothelial growth factor. J Cell Sci (1995) 108(Pt 6):2369–79. 10.1242/jcs.108.6.2369 7673356

[195] Roberts WG Palade GE. Neovasculature induced by vascular endothelial growth factor is fenestrated. Cancer Res (1997) 57:765–72. 9044858

[196] Stan RV Roberts WG Predescu D Ihida K Saucan L Ghitescu L. Immunoisolation and partial characterization of endothelial plasmalemmal vesicles (caveolae). Mol Biol Cell (1997) 8:595–605. 10.1091/mbc.8.4.595 9247641 PMC276112

[197] Roberts WG Delaat J Nagane M Huang S Cavenee WK Palade GE. Host microvasculature influence on tumor vascular morphology and endothelial gene expression. Am J Pathol (1998) 153:1239–48. 10.1016/S0002-9440(10)65668-4 PMC1853053 9777955

[198] Venneri MA De Palma M Ponzoni M Pucci F Scielzo C Zonari E. Identification of proangiogenic TIE2-expressing monocytes (TEMs) in human peripheral blood and cancer. Blood (2007) 109:5276–85. 10.1182/blood-2006-10-053504 17327411

[199] Lewis CE De Palma M Naldini L. Tie2-expressing monocytes and tumor angiogenesis: regulation by hypoxia and angiopoietin-2. Cancer Res (2007) 67:8429–32. 10.1158/0008-5472.CAN-07-1684 17875679

[200] Solinas G Germano G Mantovani A Allavena P. Tumor-associated macrophages (TAM) as major players of the cancer-related inflammation. J Leukoc Biol (2009) 86:1065–73. 10.1189/jlb.0609385 19741157

[201] Squadrito ML De Palma M. Macrophage regulation of tumor angiogenesis: implications for cancer therapy. Mol Aspects Med (2011) 32:123–45. 10.1016/j.mam.2011.04.005 21565215

[202] Sierra JR Corso S Caione L Cepero V Conrotto P Cignetti A. Tumor angiogenesis and progression are enhanced by Sema4D produced by tumor-associated macrophages. J Exp Med (2008) 205:1673–85. 10.1084/jem.20072602 PMC2442644 18559453

[203] Leek RD Landers R Fox SB Ng F Harris AL Lewis CE. Association of tumour necrosis factor alpha and its receptors with thymidine phosphorylase expression in invasive breast carcinoma. Br J Cancer (1998) 77:2246–51. 10.1038/bjc.1998.373 PMC2150409 9649140

[204] Hildenbrand R Dilger I Horlin A Stutte HJ. Urokinase and macrophages in tumour angiogenesis. Br J Cancer (1995) 72:818–23. 10.1038/bjc.1995.419 PMC2034060 7547226

[205] Chen P Huang Y Bong R Ding Y Song N Wang X. Tumor-associated macrophages promote angiogenesis and melanoma growth via adrenomedullin in a paracrine and autocrine manner. Clin Cancer Res (2011) 17:7230–9. 10.1158/1078-0432.CCR-11-1354 21994414

[206] Quaranta V Schmid MC. Macrophage-Mediated Subversion of Anti-Tumour Immunity. Cells (2019) 8(7):747. 10.3390/cells8070747 PMC6678757 31331034

[207] Boldajipour B Nelson A Krummel MF. Tumor-infiltrating lymphocytes are dynamically desensitized to antigen but are maintained by homeostatic cytokine. JCI Insight (2016) 1:e89289. 10.1172/jci.insight.89289 27942588 PMC5135268

[208] Murray PJ Allen JE Biswas SK Fisher EA Gilroy DW Goerdt S. Macrophage activation and polarization: nomenclature and experimental guidelines. Immunity (2014) 41:14–20. 10.1016/j.immuni.2014.06.008 25035950 PMC4123412

[209] Laoui D Movahedi K Van Overmeire E Van den Bossche J Schouppe E Mommer C. Tumor-associated macrophages in breast cancer: distinct subsets, distinct functions. Int J Dev Biol (2011) 55:861–7. 10.1387/ijdb.113371dl 22161841

[210] Arwert EN Harney AS Entenberg D Wang Y Sahai E Pollard JW. A Unidirectional Transition from Migratory to Perivascular Macrophage Is Required for Tumor Cell Intravasation. Cell Rep (2018) 23:1239–48. 10.1016/j.celrep.2018.04.007 PMC5946803 29719241

[211] Martinez FO Gordon S. The M1 and M2 paradigm of macrophage activation: time for reassessment. F1000prime Rep (2014) 6:13. 10.12703/P6-13 24669294 PMC3944738

[212] Mantovani A Sica A. Macrophages, innate immunity and cancer: balance, tolerance, and diversity. Curr Opin Immunol (2010) 22:231–7. 10.1016/j.coi.2010.01.009 20144856

[213] Lenzo JC Turner AL Cook AD Vlahos R Anderson GP Reynolds EC. Control of macrophage lineage populations by CSF-1 receptor and GM-CSF in homeostasis and inflammation. Immunol Cell Biol (2012) 90:429–40. 10.1038/icb.2011.58 21727904

[214] Kowal J Kornete M Joyce JA. Re-education of macrophages as a therapeutic strategy in cancer. Immunotherapy (2019) 11:677–89. 10.2217/imt-2018-0156 31088236

[215] Petty AJ Li A Wang X Dai R Heyman B Hsu D. Hedgehog signaling promotes tumor-associated macrophage polarization to suppress intratumoral CD8+ T cell recruitment. J Clin Invest (2019) 129:5151–62. 10.1172/JCI128644 PMC6877305 31638600

[216] Guirnalda P Wood L Goenka R Crespo J Paterson Y. Interferon gamma-induced intratumoral expression of CXCL9 alters the local distribution of T cells following immunotherapy with Listeria monocytogenes. Oncoimmunology (2013) 2:e25752. 10.4161/onci.25752 24083082 PMC3782529

[217] Vilgelm AE Richmond A. Chemokines Modulate Immune Surveillance in Tumorigenesis, Metastasis, and Response to Immunotherapy. Front Immunol (2019) 10:333. 10.3389/fimmu.2019.00333 30873179 PMC6400988

[218] Harlin H Meng Y Peterson AC Zha Y Tretiakova M Slingluff C. Chemokine expression in melanoma metastases associated with CD8+ T-cell recruitment. Cancer Res (2009) 69:3077–85. 10.1158/0008-5472.CAN-08-2281 PMC3886718 19293190

[219] Nagarsheth N Wicha MS Zou W. Chemokines in the cancer microenvironment and their relevance in cancer immunotherapy. Nat Rev Immunol (2017) 17:559–72. 10.1038/nri.2017.49 PMC5731833 28555670

[220] Karin N Razon H. Chemokines beyond chemo-attraction: CXCL10 and its significant role in cancer and autoimmunity. Cytokine (2018) 109:24–8. 10.1016/j.cyto.2018.02.012 29449068

[221] Hensbergen PJ Wijnands PG Schreurs MW Scheper RJ Willemze R Tensen CP. The CXCR3 targeting chemokine CXCL11 has potent antitumor activity in vivo involving attraction of CD8+ T lymphocytes but not inhibition of angiogenesis. J Immunother (2005) 28:343–51. 10.1097/01.cji.0000165355.26795.27 16000952

[222] Lanitis E Dangaj D Irving M Coukos G. Mechanisms regulating T-cell infiltration and activity in solid tumors. Ann Oncol (2017) 28:xii18–32. 10.1093/annonc/mdx238 29045511

[223] Batlle E Massague J. Transforming Growth Factor-beta Signaling in Immunity and Cancer. Immunity (2019) 50:924–40. 10.1016/j.immuni.2019.03.024 PMC7507121 30995507

[224] Colak S Ten Dijke P. Targeting TGF-beta Signaling in Cancer. Trends Cancer (2017) 3:56–71. 10.1016/j.trecan.2016.11.008 28718426

[225] Pickup MW Owens P Moses HL. TGF-beta, Bone Morphogenetic Protein, and Activin Signaling and the Tumor Microenvironment. Cold Spring Harbor Perspect Biol (2017) 9:a022285. 10.1101/cshperspect.a022285 PMC5411701 28062564

[226] Kelly A Gunaltay S McEntee CP Shuttleworth EE Smedley C Houston SA. Human monocytes and macrophages regulate immune tolerance via integrin alphavbeta8-mediated TGFbeta activation. J Exp Med (2018) 215:2725–36. 10.1084/jem.20171491 PMC6219736 30355614

[227] Tauriello DVF Palomo-Ponce S Stork D Berenguer-Llergo A Badia-Ramentol J Iglesias M. TGFbeta drives immune evasion in genetically reconstituted colon cancer metastasis. Nature (2018) 554:538–43. 10.1038/nature25492 29443964

[228] Mariathasan S Turley SJ Nickles D Castiglioni A Yuen K Wang Y. TGFbeta attenuates tumour response to PD-L1 blockade by contributing to exclusion of T cells. Nature (2018) 554:544–8. 10.1038/nature25501 PMC6028240 29443960

[229] Pitt LA Tikhonova AN Hu H Trimarchi T King B Gong Y. CXCL12-Producing Vascular Endothelial Niches Control Acute T Cell Leukemia Maintenance. Cancer Cell (2015) 27:755–68. 10.1016/j.ccell.2015.05.002 PMC4461838 26058075

[230] Feig C Jones JO Kraman M Wells RJ Deonarine A Chan DS. Targeting CXCL12 from FAP-expressing carcinoma-associated fibroblasts synergizes with anti-PD-L1 immunotherapy in pancreatic cancer. Proc Natl Acad Sci USA (2013) 110:20212–7. 10.1073/pnas.1320318110 PMC3864274 24277834

[231] Boimel PJ Smirnova T Zhou ZN Wyckoff J Park H Coniglio SJ. Contribution of CXCL12 secretion to invasion of breast cancer cells. Breast Cancer Res BCR (2012) 14:R23. 10.1186/bcr3108 22314082 PMC3496141

[232] Rao S Sengupta R Choe EJ Woerner BM Jackson E Sun T. CXCL12 mediates trophic interactions between endothelial and tumor cells in glioblastoma. PLoS One (2012) 7:e33005. 10.1371/journal.pone.0033005 22427929 PMC3299723

[233] Mosadegh B Saadi W Wang SJ Jeon NL. Epidermal growth factor promotes breast cancer cell chemotaxis in CXCL12 gradients. Biotechnol Bioeng (2008) 100:1205–13. 10.1002/bit.21851 18553401

[234] Orimo A Gupta PB Sgroi DC Arenzana-Seisdedos F Delaunay T Naeem R. Stromal fibroblasts present in invasive human breast carcinomas promote tumor growth and angiogenesis through elevated SDF-1/CXCL12 secretion. Cell (2005) 121:335–48. 10.1016/j.cell.2005.02.034 15882617

[235] Joyce JA Fearon DT. T cell exclusion, immune privilege, and the tumor microenvironment. Science (2015) 348:74–80. 10.1126/science.aaa6204 25838376

[236] Chen IX Chauhan VP Posada J Ng MR Wu MW Adstamongkonkul P. Blocking CXCR4 alleviates desmoplasia, increases T-lymphocyte infiltration, and improves immunotherapy in metastatic breast cancer. Proc Natl Acad Sci USA (2019) 116:4558–66. 10.1073/pnas.1815515116 PMC6410779 30700545

[237] Cronin PA Wang JH Redmond HP. Hypoxia increases the metastatic ability of breast cancer cells via upregulation of CXCR4. BMC Cancer (2010) 10:225. 10.1186/1471-2407-10-225 20492653 PMC2880996

[238] Taromi S Kayser G Catusse J von Elverfeldt D Reichardt W Braun F. CXCR4 antagonists suppress small cell lung cancer progression. Oncotarget (2016) 7:85185–95. 10.18632/oncotarget.13238 PMC5356728 27835905

[239] Ward PS Thompson CB. Metabolic reprogramming: a cancer hallmark even warburg did not anticipate. Cancer Cell (2012) 21:297–308. 10.1016/j.ccr.2012.02.014 22439925 PMC3311998

[240] Filipp FV Ratnikov B De Ingeniis J Smith JW Osterman AL Scott DA. Glutamine-fueled mitochondrial metabolism is decoupled from glycolysis in melanoma. Pigment Cell Melanoma Res (2012) 25:732–9. 10.1111/pcmr.12000 PMC3639292 22846158

[241] Fan J Kamphorst JJ Mathew R Chung MK White E Shlomi T. Glutamine-driven oxidative phosphorylation is a major ATP source in transformed mammalian cells in both normoxia and hypoxia. Mol Syst Biol (2013) 9:712. 10.1038/msb.2013.65 24301801 PMC3882799

[242] Son J Lyssiotis CA Ying H Wang X Hua S Ligorio M. Glutamine supports pancreatic cancer growth through a KRAS-regulated metabolic pathway. Nature (2013) 496:101–5. 10.1038/nature12040 PMC3656466 23535601

[243] Ying H Kimmelman AC Lyssiotis CA Hua S Chu GC Fletcher-Sananikone E. Oncogenic Kras maintains pancreatic tumors through regulation of anabolic glucose metabolism. Cell (2012) 149:656–70. 10.1016/j.cell.2012.01.058 PMC3472002 22541435

[244] Carvalho KC Cunha IW Rocha RM Ayala FR Cajaiba MM Begnami MD. GLUT1 expression in malignant tumors and its use as an immunodiagnostic marker. Clinics (Sao Paulo) (2011) 66:965–72. 10.1590/S1807-59322011000600008 PMC3129958 21808860

[245] Kaira K Sunose Y Arakawa K Ogawa T Sunaga N Shimizu K. Prognostic significance of L-type amino-acid transporter 1 expression in surgically resected pancreatic cancer. Br J Cancer (2012) 107:632–8. 10.1038/bjc.2012.310 PMC3419959 22805328

[246] Kaira K Nakamura K Hirakawa T Imai H Tominaga H Oriuchi N. Prognostic significance of L-type amino acid transporter 1 (LAT1) expression in patients with ovarian tumors. Am J Transl Res (2015) 7:1161–71. PMC4532748 26279759

[247] Wang Q Beaumont KA Otte NJ Font J Bailey CG van Geldermalsen M. Targeting glutamine transport to suppress melanoma cell growth. Int J Cancer (2014) 135:1060–71. 10.1002/ijc.28749 24531984

[248] Ueda SM Yap KL Davidson B Tian Y Murthy V Wang TL. Expression of Fatty Acid Synthase Depends on NAC1 and Is Associated with Recurrent Ovarian Serous Carcinomas. J Oncol (2010) 2010:285191. 10.1155/2010/285191 20508725 PMC2873657

[249] Innocenzi D Alo PL Balzani A Sebastiani V Silipo V La Torre G. Fatty acid synthase expression in melanoma. J Cutan Pathol (2003) 30:23–8. 10.1034/j.1600-0560.2003.300104.x 12534800

[250] Bian Y Yu Y Wang S Li L. Up-regulation of fatty acid synthase induced by EGFR/ERK activation promotes tumor growth in pancreatic cancer. Biochem Biophys Res Commun (2015) 463:612–7. 10.1016/j.bbrc.2015.05.108 26043686

[251] O’Flanagan CH Bowers LW Hursting SD. A weighty problem: metabolic perturbations and the obesity-cancer link. Horm Mol Biol Clin Investig (2015) 23:47–57. 10.1515/hmbci-2015-0022 PMC4839982 26167982

[252] Pearce EL Walsh MC Cejas PJ Harms GM Shen H Wang LS. Enhancing CD8 T-cell memory by modulating fatty acid metabolism. Nature (2009) 460:103–7. 10.1038/nature08097 PMC2803086 19494812

[253] Ringel AE Drijvers JM Baker GJ Catozzi A Garcia-Canaveras JC Gassaway BM. Obesity Shapes Metabolism in the Tumor Microenvironment to Suppress Anti-Tumor Immunity. Cell (2020) 183(7):1848–66.e26. 10.1016/j.cell.2020.11.009 PMC8064125 33301708

[254] Lin R Zhang H Yuan Y He Q Zhou J Li S. Fatty Acid Oxidation Controls CD8(+) Tissue-Resident Memory T-cell Survival in Gastric Adenocarcinoma. Cancer Immunol Res (2020) 8:479–92. 10.1158/2326-6066.CIR-19-0702 32075801

[255] Li W Wei Z Liu Y Li H Ren R Tang Y. Increased 18F-FDG uptake and expression of Glut1 in the EMT transformed breast cancer cells induced by TGF-beta. Neoplasma (2010) 57:234–40. 10.4149/neo_2010_03_234 20353274

[256] Oh S Kim H Nam K Shin I. Glut1 promotes cell proliferation, migration and invasion by regulating epidermal growth factor receptor and integrin signaling in triple-negative breast cancer cells. BMB Rep (2017) 50:132–7. 10.5483/BMBRep.2017.50.3.189 PMC5422025 27931517

[257] Sullivan WJ Mullen PJ Schmid EW Flores A Momcilovic M Sharpley MS. Extracellular Matrix Remodeling Regulates Glucose Metabolism through TXNIP Destabilization. Cell (2018) 175:117–32 e21. 10.1016/j.cell.2018.08.017 30197082 PMC6151140

[258] Singer K Kastenberger M Gottfried E Hammerschmied CG Buttner M Aigner M. Warburg phenotype in renal cell carcinoma: high expression of glucose-transporter 1 (GLUT-1) correlates with low CD8(+) T-cell infiltration in the tumor. Int J Cancer (2011) 128:2085–95. 10.1002/ijc.25543 20607826

[259] Ottensmeier CH Perry KL Harden EL Stasakova J Jenei V Fleming J. Upregulated Glucose Metabolism Correlates Inversely with CD8+ T-cell Infiltration and Survival in Squamous Cell Carcinoma. Cancer Res (2016) 76:4136–48. 10.1158/0008-5472.CAN-15-3121 27206847

[260] Grohmann U Fallarino F Bianchi R Vacca C Orabona C Belladonna ML. Tryptophan catabolism in nonobese diabetic mice. Adv Exp Med Biol (2003) 527:47–54. 10.1007/978-1-4615-0135-0_5 15206715

[261] Grohmann U Fallarino F Puccetti P. Tolerance, DCs and tryptophan: much ado about IDO. Trends Immunol (2003) 24:242–8. 10.1016/S1471-4906(03)00072-3 12738417

[262] Fallarino F Grohmann U Vacca C Orabona C Spreca A Fioretti MC. T cell apoptosis by kynurenines. Adv Exp Med Biol (2003) 527:183–90. 10.1007/978-1-4615-0135-0_21 15206731

[263] Sucher R Kurz K Weiss G Margreiter R Fuchs D Brandacher G. IDO-Mediated Tryptophan Degradation in the Pathogenesis of Malignant Tumor Disease. Int J Tryptophan Res (2010) 3:113–20. 10.4137/IJTR.S4157 PMC3195236 22084593

[264] Yan H Dong M Liu X Shen Q He D Huang X. Multiple myeloma cell-derived IL-32gamma increases the immunosuppressive function of macrophages by promoting indoleamine 2,3-dioxygenase (IDO) expression. Cancer Lett (2019) 446:38–48. 10.1016/j.canlet.2019.01.012 30660652

[265] Liu WL Lin YH Xiao H Xing S Chen H Chi PD. Epstein-Barr virus infection induces indoleamine 2,3-dioxygenase expression in human monocyte-derived macrophages through p38/mitogen-activated protein kinase and NF-kappaB pathways: impairment in T cell functions. J Virol (2014) 88:6660–71. 10.1128/JVI.03678-13 PMC4054364 24696473

[266] Baitsch L Baumgaertner P Devevre E Raghav SK Legat A Barba L. Exhaustion of tumor-specific CD8(+) T cells in metastases from melanoma patients. J Clin Invest (2011) 121:2350–60. 10.1172/JCI46102 PMC3104769 21555851

[267] Giordano M Henin C Maurizio J Imbratta C Bourdely P Buferne M. Molecular profiling of CD8 T cells in autochthonous melanoma identifies Maf as driver of exhaustion. EMBO J (2015) 34:2042–58. 10.15252/embj.201490786 PMC4551351 26139534

[268] Stromnes IM Schmitt TM Hulbert A Brockenbrough JS Nguyen H Cuevas C. T Cells Engineered against a Native Antigen Can Surmount Immunologic and Physical Barriers to Treat Pancreatic Ductal Adenocarcinoma. Cancer Cell (2015) 28:638–52. 10.1016/j.ccell.2015.09.022 PMC4724422 26525103

[269] Zippelius A Batard P Rubio-Godoy V Bioley G Lienard D Lejeune F. Effector function of human tumor-specific CD8 T cells in melanoma lesions: a state of local functional tolerance. Cancer Res (2004) 64:2865–73. 10.1158/0008-5472.CAN-03-3066 15087405

[270] Blank CU Haining WN Held W Hogan PG Kallies A Lugli E. Defining ‘T cell exhaustion’. Nat Rev Immunol (2019) 19:665–74. 10.1038/s41577-019-0221-9 PMC7286441 31570879

[271] Blackburn SD Shin H Haining WN Zou T Workman CJ Polley A. Coregulation of CD8+ T cell exhaustion by multiple inhibitory receptors during chronic viral infection. Nat Immunol (2009) 10:29–37. 10.1038/ni.1679 19043418 PMC2605166

[272] Wherry EJ Kurachi M. Molecular and cellular insights into T cell exhaustion. Nat Rev Immunol (2015) 15:486–99. 10.1038/nri3862 PMC4889009 26205583

[273] Bolouri H Young M Beilke J Johnson R Fox B Huang L. Integrative network modeling reveals mechanisms underlying T cell exhaustion. Sci Rep (2020) 10:1915. 10.1038/s41598-020-58600-8 32024856 PMC7002445

[274] Wing K Onishi Y Prieto-Martin P Yamaguchi T Miyara M Fehervari Z. CTLA-4 control over Foxp3+ regulatory T cell function. Science (2008) 322:271–5. 10.1126/science.1160062 18845758

[275] Chikuma S. CTLA-4, an Essential Immune-Checkpoint for T-Cell Activation. Curr Top Microbiol Immunol (2017) 410:99–126. 10.1007/82_2017_61 28900679

[276] Sheppard KA Fitz LJ Lee JM Benander C George JA Wooters J. PD-1 inhibits T-cell receptor induced phosphorylation of the ZAP70/CD3zeta signalosome and downstream signaling to PKCtheta. FEBS Lett (2004) 574:37–41. 10.1016/j.febslet.2004.07.083 15358536

[277] Francisco LM Salinas VH Brown KE Vanguri VK Freeman GJ Kuchroo VK. PD-L1 regulates the development, maintenance, and function of induced regulatory T cells. J Exp Med (2009) 206:3015–29. 10.1084/jem.20090847 PMC2806460 20008522

[278] Huang CT Workman CJ Flies D Pan X Marson AL Zhou G. Role of LAG-3 in regulatory T cells. Immunity (2004) 21:503–13. 10.1016/j.immuni.2004.08.010 15485628

[279] Johnston RJ Comps-Agrar L Hackney J Yu X Huseni M Yang Y. The immunoreceptor TIGIT regulates antitumor and antiviral CD8(+) T cell effector function. Cancer Cell (2014) 26:923–37. 10.1016/j.ccell.2014.10.018 25465800

[280] Singer M Wang C Cong L Marjanovic ND Kowalczyk MS Zhang H. A Distinct Gene Module for Dysfunction Uncoupled from Activation in Tumor-Infiltrating T Cells. Cell (2016) 166:1500–11. 10.1016/j.cell.2016.08.052 PMC5019125 27610572

[281] Mihic-Probst D Reinehr M Dettwiler S Kolm I Britschgi C Kudura K. The role of macrophages type 2 and T-regs in immune checkpoint inhibitor related adverse events. Immunobiology (2020) 225:152009. 10.1016/j.imbio.2020.152009 32962812

[282] Huang YK Wang M Sun Y Di Costanzo N Mitchell C Achuthan A. Macrophage spatial heterogeneity in gastric cancer defined by multiplex immunohistochemistry. Nat Commun (2019) 10:3928. 10.1038/s41467-019-11788-4 31477692 PMC6718690

[283] Zhu Y Knolhoff BL Meyer MA Nywening TM West BL Luo J. CSF1/CSF1R blockade reprograms tumor-infiltrating macrophages and improves response to T-cell checkpoint immunotherapy in pancreatic cancer models. Cancer Res (2014) 74:5057–69. 10.1158/0008-5472.CAN-13-3723 PMC4182950 25082815

[284] Zhang Y Dong X Bai L Shang X Zeng Y. MUC1-induced immunosuppression in colon cancer can be reversed by blocking the PD1/PDL1 signaling pathway. Oncol Lett (2020) 20:317. 10.3892/ol.2020.12180 PMC7590440 33133253

[285] Dutsch-Wicherek M Kazmierczak W. Creation of a suppressive microenvironment by macrophages and cancer-associated fibroblasts. Front Biosci (2013) 18:1003–16. 10.2741/4159 23747863

[286] Li L Han L Sun F Zhou J Ohaegbulam KC Tang X. NF-kappaB RelA renders tumor-associated macrophages resistant to and capable of directly suppressing CD8(+) T cells for tumor promotion. Oncoimmunology (2018) 7:e1435250. 10.1080/2162402X.2018.1435250 29872577 PMC5980414

[287] Wherry EJ Ha SJ Kaech SM Haining WN Sarkar S Kalia V. Molecular signature of CD8+ T cell exhaustion during chronic viral infection. Immunity (2007) 27:670–84. 10.1016/j.immuni.2007.09.006 17950003

[288] Coffelt SB Chen YY Muthana M Welford AF Tal AO Scholz A. Angiopoietin 2 stimulates TIE2-expressing monocytes to suppress T cell activation and to promote regulatory T cell expansion. J Immunol (2011) 186:4183–90. 10.4049/jimmunol.1002802 21368233

[289] Kado T Nawaz A Takikawa A Usui I Tobe K. Linkage of CD8(+) T cell exhaustion with high-fat diet-induced tumourigenesis. Sci Rep (2019) 9:12284. 10.1038/s41598-019-48678-0 31439906 PMC6706391

[290] Landskron G De la Fuente M Thuwajit P Thuwajit C Hermoso MA. Chronic inflammation and cytokines in the tumor microenvironment. J Immunol Res (2014) 2014:149185. 10.1155/2014/149185 24901008 PMC4036716

[291] Jiang Y Li Y Zhu B. T-cell exhaustion in the tumor microenvironment. Cell Death Dis (2015) 6:e1792. 10.1038/cddis.2015.162 26086965 PMC4669840

[292] Chen W Jin W Hardegen N Lei KJ Li L Marinos N. Conversion of peripheral CD4+CD25- naive T cells to CD4+CD25+ regulatory T cells by TGF-beta induction of transcription factor Foxp3. J Exp Med (2003) 198:1875–86. 10.1084/jem.20030152 PMC2194145 14676299

[293] Sosa MS Bragado P Aguirre-Ghiso JA. Mechanisms of disseminated cancer cell dormancy: an awakening field. Nat Rev Cancer (2014) 14:611–22. 10.1038/nrc3793 PMC4230700 25118602

[294] Grenga I Kwilas AR Donahue RN Farsaci B Hodge JW. Inhibition of the angiopoietin/Tie2 axis induces immunogenic modulation, which sensitizes human tumor cells to immune attack. J Immunother Cancer (2015) 3:52. 10.1186/s40425-015-0096-7 26579226 PMC4647578

[295] Arlauckas SP Garris CS Kohler RH Kitaoka M Cuccarese MF Yang KS. In vivo imaging reveals a tumor-associated macrophage-mediated resistance pathway in anti-PD-1 therapy. Sci Transl Med (2017) 9:eaal3604. 10.1126/scitranslmed.aal3604 PMC5734617 28490665

[296] Postow MA Callahan MK Wolchok JD. Immune Checkpoint Blockade in Cancer Therapy. J Clin Oncol (2015) 33(17)1974–82. 10.1200/JCO.2014.59.4358 PMC4980573 25605845

[297] Georganaki M van Hooren L Dimberg A. Vascular Targeting to Increase the Efficiency of Immune Checkpoint Blockade in Cancer. Front Immunol (2018) 9:3081. 10.3389/fimmu.2018.03081 30627131 PMC6309238

[298] Lucignani G Ottobrini L Martelli C Rescigno M Clerici M. Molecular imaging of cell-mediated cancer immunotherapy. Trends Biotechnol (2006) 24:410–8. 10.1016/j.tibtech.2006.07.003 16870284

[299] Coste A Oktay MH Condeelis JS Entenberg D. Intravital Imaging Techniques for Biomedical and Clinical Research. Cytometry A (2019) 97(5):448–57. 10.1002/cyto.a.23963 PMC7210060 31889408

[300] Krekorian M Fruhwirth GO Srinivas M Figdor CG Heskamp S Witney TH. Imaging of T-cells and their responses during anti-cancer immunotherapy. Theranostics (2019) 9:7924–47. 10.7150/thno.37924 PMC6814447 31656546

